# A review of 3D first-pass, whole-heart, myocardial perfusion cardiovascular magnetic resonance

**DOI:** 10.1186/s12968-015-0162-9

**Published:** 2015-08-01

**Authors:** Merlin J. Fair, Peter D. Gatehouse, Edward V. R. DiBella, David N. Firmin

**Affiliations:** National Heart & Lung Institute, Imperial College London, London, UK; Cardiovascular Magnetic Resonance Unit, Royal Brompton Hospital, Sydney Street, London, SW3 6NP UK; Utah Center for Advanced Imaging Research, University of Utah, Salt Lake City, UT USA

**Keywords:** Myocardial perfusion, 3D, Whole heart, Cardiovascular magnetic resonance

## Abstract

A comprehensive review is undertaken of the methods available for 3D whole-heart first-pass perfusion (FPP) and their application to date, with particular focus on possible acceleration techniques. Following a summary of the parameters typically desired of 3D FPP methods, the review explains the mechanisms of key acceleration techniques and their potential use in FPP for attaining 3D acquisitions. The mechanisms include rapid sequences, non-Cartesian k-space trajectories, reduced k-space acquisitions, parallel imaging reconstructions and compressed sensing. An attempt is made to explain, rather than simply state, the varying methods with the hope that it will give an appreciation of the different components making up a 3D FPP protocol. Basic estimates demonstrating the required total acceleration factors in typical 3D FPP cases are included, providing context for the extent that each acceleration method can contribute to the required imaging speed, as well as potential limitations in present 3D FPP literature. Although many 3D FPP methods are too early in development for the type of clinical trials required to show any clear benefit over current 2D FPP methods, the review includes the small but growing quantity of clinical research work already using 3D FPP, alongside the more technical work. Broader challenges concerning FPP such as quantitative analysis are not covered, but challenges with particular impact on 3D FPP methods, particularly with regards to motion effects, are discussed along with anticipated future work in the field.

## Introduction

Detection of coronary artery disease (CAD) through examination of dynamically contrast-enhanced myocardial perfusion cardiovascular magnetic resonance (CMR) is well established clinically [1, 2], following its first demonstrations in 1990 [3]. Dynamic contrast enhancement (DCE), here called first-pass perfusion (FPP), has shown high diagnostic accuracy [4] and compares favourably with other modalities as a “gate-keeper” to invasive coronary x-ray angiography [5]. Despite this, there are a multitude of desired properties in an ideal FPP protocol that CMR is currently unable to simultaneously meet with standard imaging speeds. In particular is the extension of FPP protocols from 2D non-contiguous coverage of the left ventricle (LV) to 3D whole-heart imaging, which has been hypothesised as a way of increasing the competitiveness of CMR for perfusion imaging [6]. Whilst there is debate over the clinical utility of 3D FPP when coverage is at the expense of other imaging parameters (discussed further in section *Imaging parameters for FPP*), there is interest in its potential; for example, possible increased confidence by obtaining more slices over the same cardiac regions and the slices being all at the same cardiac phase. There has therefore been a recent surge in publications on 3D FPP (see Table 1), and with it increasing application of extreme acceleration to FPP.

**Table 1:** Overview of technical developments in 3D whole-heart first-pass perfusion

Lead Author	Year	Reconstruction Method	Trajectory	Other Stated Efficiencies*	US Factor (Nominal)	US Factor (True)	Resolution/mm	Acquisition Window/ms	Stress Agent	Free Breathing†	Field Strength/T
Shin [70]	2008	TSENSE	Cartesian	-	6	6	3.0 × 4.5 × 10.0	304	No	No	3
Shin [19]	2010	TSENSE	Cartesian	-	6	6	4.5 × 6.7 × 10.0^a^/2.8 × 4.2 × 10.0^b^	116-145^a^/254-305^b^	No	No	3
Manka [79]◊	2011	k-t SENSE	Cartesian	Partial Fourier	*NS*	6.3	2.3 × 2.3 × 10.0	200	Yes	No	3
Vitanis [81]	2011	k-t PCA (compartment based)	Cartesian	Elliptical Shutter (75 %); Partial Fourier (75 %)	10	5.6 - 7.5	2.3 × 2.3 × 10.0	225	Yes	No	3
DiBella [25]	2012	CS (temporal)	Radial	-	~14‡	~14‡	2.2 × 2.2 × 8.0	310	No	No	3
Manka [125] ◊	2012	k-t PCA	Cartesian	Partial Fourier	10	7	2.3 × 2.3 × 10.0	*NS*	Yes	No	1.5
Jogiya [126] ◊	2012	k-t PCA	Cartesian	Partial Fourier	10	7	2.3 × 2.3 × 5.0	*NS*	Yes	No	3
Chen [44]	2012	CS (spatio- temporal)	Radial	Partial Fourier (75 %)	~9-11‡	~9-11‡	(1.8-2.8) × (1.8-2.8) × (6.0-10.0)	300	No	No	3
Shin [39]	2013	k-t SENSE	Spiral	-	5	5	2.4 × 2.4 × 9.0^~^	230	No	No	1.5
Giri [24]	2014	TWIST (GRAPPA)	Cartesian	2D Partial Fourier§ (87.5 %/87.5 %)	3	3	2.2 × 2.8 × 8.0	300-380	No	No	1.5
Motwani [56]	2014	k-t PCA	Cartesian	2D Partial Fourier (70 %/70 %)	10	7	2.3 × 2.3 × 5.0	192	Yes	No	3
Schmidt [58]	2014	k-t PCA (motion-corrected)	Cartesian	Elliptical Shutter; 3D Partial Fourier (62.5 %/75 %/75 %)	10	*NS*	2.3 × 2.3 × 10.0	205-225	No	Yes	3
Akçakaya [105]	2014	CS (localised constraints)	Cartesian	Elliptical Shutter (75 %)	10	10	2.3 × 2.3 × 10.0	250	No	Both	1.5
Jogiya [57]◊	2014^e^	k-t PCA	Cartesian	Elliptical Shutter; 2D Partial Fourier (75 %/75 %)	10	7	2.3 × 2.3 × 5.0	191	Yes	No	3
Jogiya [21]	2014	k-t PCA	Cartesian	Elliptical Shutter; 3D Partial Fourier (NS/75 %/75 %)	10	7	2.3 × 2.3 × 5.0	191	Yes	No	3
Wang [45]	2014^e^	CS (spatio-temporal)	Cartesian	Partial Fourier (83 %)	11	11	(2.0-2.4) × (2.0-2.4) × (4.0-6.0)	255	No	No	3
Manka [130]◊	2015^e^	k-t PCA	Cartesian	Elliptical Shutter; 2D Partial Fourier (75 %/75 %)	10	7	2.3 × 2.3 × 5.0	200	Yes	No	3

◊Clinically relevant/tested techniques – see Table 2 for details

*Zero padding typically not stated when applied, so not included in the table

†Imaging during breath-hold assumed in cases where literature did not state

‡US factor estimated from number of radial projections and number of readouts: N_Nyquist_ = (π/2) * N_readouts_, N.b. using this definition radial trajectories require π/2 greater acceleration to be equivalent to a Cartesian acquisition time

§Only applied in cases of high HR

^a^Systolic acquisition values

^b^Diastolic acquisition values

^~^Nominal spatial resolution can be affected by off-resonance errors in long spiral readouts

^e^Date of early online publication, yet to be published fully at time of print

NB The acquisition window as far as possible is the pure image data acquisition time not including saturation recovery delay pre-imaging as that is not normally motion-sensitive

*Abbreviations*: *US* undersampling; *NS* not stated; others as defined in text

The purpose of this review is exploration of this wide range of current and potential techniques for achieving 3D FPP, in particular the acceleration of data acquisition. The characteristics of ideal FPP methods are reviewed first, with some reflection on the issues governing typical multi-slice 2D FPP. This follows into a justification for the approximate acceleration required for translation to 3D, before a review and explanation of two main categories of acceleration methods applicable to 3D FPP. These are referred to as pulse sequence modification and sub-Nyquist reconstruction techniques, and include non-Cartesian k-space trajectories, k-space efficiencies, and multiple varieties of parallel imaging and compressed sensing. Having presented these methods concurrent with examples in 3D FPP literature, the smaller amount of clinical research is summarised. The issues arising with applying such acceleration techniques to FPP are then examined before finally discussing future considerations and requirements.

### Imaging parameters for FPP

As with most medical imaging, the ideal parameters for 2D or 3D FPP are many, interdependent and often contradictory, causing a ‘trade-off’ in any realistic setting. They can broadly be broken down into the following areas: High spatial and temporal resolution, high signal-to-noise ratio (SNR) and coverage of the LV that supports the clinical purpose effectively. Unlike some other CMR applications, however, reliability is also critical for FPP of gadolinium-based contrast agents (GBCA) because reacquisition is impracticable.

Fine spatial resolution is required in-plane in order to adequately resolve the transmurality and extent of perfusion defects. It is also important for the problematic dark-rim artefact (DRA) [7], which frequently confounds imaging of perfusion defects. The DRA is partly caused by a Gibbs truncation effect at the sharp signal changes between the subendocardial myocardium and brighter contrast-enhanced blood-pool, and has been shown to be reduced at increased spatial resolutions [8]. For visual analysis of most defects, an isotropic in-plane resolution of 2 mm to 2.5 mm is generally deemed a sufficient balance in the trade-off against image acquisition duration and other parameters. Through-plane resolution is less important for clinical evaluation because the slice (or for 3D, 2nd phase-encoding) direction is typically along the long-axis of the heart. The impact of Gibbs artefacts in the through-plane direction has however so far undergone little investigation for 3D FPP [9]. An acquired slice-direction resolution of around 10 mm is typical in FPP, but in 3D imaging the final slice resolution is sometimes interpolated from coarser acquisitions, as will be reviewed later.

The temporal resolution of FPP imaging, i.e. imaging each slice every single cardiac cycle (“single-RR”) or alternate cycles (“alternate-RR”), is another important consideration without a clear consensus and to some extent depending on the clinical application [10]. Acquiring single-RR multi-slice 2D arguably might increase diagnostic confidence in some situations. On the other hand, alternate-RR can deliver greater myocardial coverage and with careful setup has potential to avoid imaging during phases of rapid cardiac motion [1, 11]. To date the topic of 3D FPP has entirely used single-RR, presumably due to the risk of data inconsistency if split over two cycles. This would be a more extreme version of some 3D FPP methods reviewed below in which some aspects of their raw data are potentially shared between adjoining cardiac cycles. However, some type of dual-slab alternate-RR method may have potential for reducing the toughest constraints on single-RR 3D FPP.

Myocardial SNR is of increased concern in the topic of 3D FPP due to the potential for SNR loss caused by many of the acceleration techniques presented here. The related contrast-to-noise ratio (CNR) is required to be high in distinguishing a perfusion defect from the normal tissue, especially as a defect may be only a mild limitation of blood supply. However CNR depends on many other factors such as contrast agent dose, T1 sensitivity, etc (see, for example, reference [10]).

Full coverage of the LV is often cited as a reason for investigating 3D FPP, but this is subject to the following discussion. Other perfusion modalities such as SPECT and PET, whilst providing lower resolutions, usually offer full coverage of the LV, whereas conventional CMR FPP acquires typically 3 or 4 equi-distant short-axis slices along the LV – although long-axis myocardial motion modulates their true myocardial coverage. There is some debate over the clinical utility of coverage of the whole LV [1, 12], hereafter referred to as “whole-heart” coverage, particularly when at the expense of other factors such as spatial resolution [13]. 2D FPP has already been proven a reliable method of investigating CAD, showing high accuracy in comparison to other techniques, as is reflected in multiple clinical guidelines. It has been shown that coverage with 3 or 4 short-axis slices is ample for clinical accuracy [1]. However, some potential clinical advantages of full coverage have been proposed. Most important of these is to assess the “ischaemic burden”, due to its link to survival prognosis [14]. Secondly, it may improve confidence that no defects have been missed, although the coverage by 2D multi-slice has been shown sufficient as discussed. Thirdly, it may also assist in distinguishing between DRAs and true perfusion defects, as 3D may enable improved tracking of the hypointense region through-plane for discriminating between the two, in some situations employing knowledge of typical coronary territories as already used where appropriate in 2D FPP clinical work [15].

Continuous coverage (even if not whole-heart) supports the use of 3D imaging, with a potentially strong benefit that all the images in each cycle are at the same cardiac phase and respiratory phase, even if both are liable to intra-shot motion artefacts. 3D imaging acquires the raw data (k-space) with additional repeated acquisitions of phase-encoding (N_partitions_) used to collect the third dimension, called slice or partition encoding. Along with providing contiguous coverage, this delivers a fundamental increase in SNR. From Edelstein et al. [16], keeping all other factors the same, it can be derived that the SNR in 3D compared to its 2D equivalent is

$$ SN{R}_{3D}=SN{R}_{2D}\times \sqrt{N_{partitions}}. $$

A limitation to this equation is that it does not account for alterations in saturation caused by the slab RF excitation pulses compared to multi-slice. No direct measurement of this predicted SNR gain in 3D FPP has been performed, largely due to the modification of SNR by the acceleration methods required for 3D.

Whilst attempting to achieve the above parameters the final factor of note is in achieving reliability of the protocol. The imaging of the first-pass of GBCA makes repetition of acquisitions impracticable due to dose limits and wash out time, therefore making reliability key. Practically, a requirement for breath-hold, in support of the acceleration methods discussed later, introduces unreliability over whether the breath-hold is maintained during the key frames of contrast arrival, although the importance of this is debated. A requirement of breath-hold or gentle breathing is made more difficult by the potential impact of adenosine or other stressors on respiratory motion. The additional effect of misgating on acceleration methods is another concern for reliability.

### Requirement for acceleration in 3D FPP

As has been mentioned, the above requirements cannot normally be achieved simultaneously, with spatial resolution, temporal resolution, LV coverage, reliability and SNR being traded off against each other. To gain an understanding of the shortfall of a basic imaging sequence in achieving all these properties, timings of a 3D fast low flip-angle spoiled gradient-echo (FLASH) sequence were calculated to illustrate potential optimal acquisition times (see Appendix). Despite using a sequence based on timing minimisation rather than image quality, this gives an acquisition time of around 2.8 s to acquire data of the whole-heart with the proposed parameters; this illustrates the scale of the challenge because FPP imaging requires at least alternate-cycle imaging of GBCA distribution, so the usual “segmented” methods for accumulating resolution over multiple cardiac cycles cannot be applied.

**Fig. 1:**
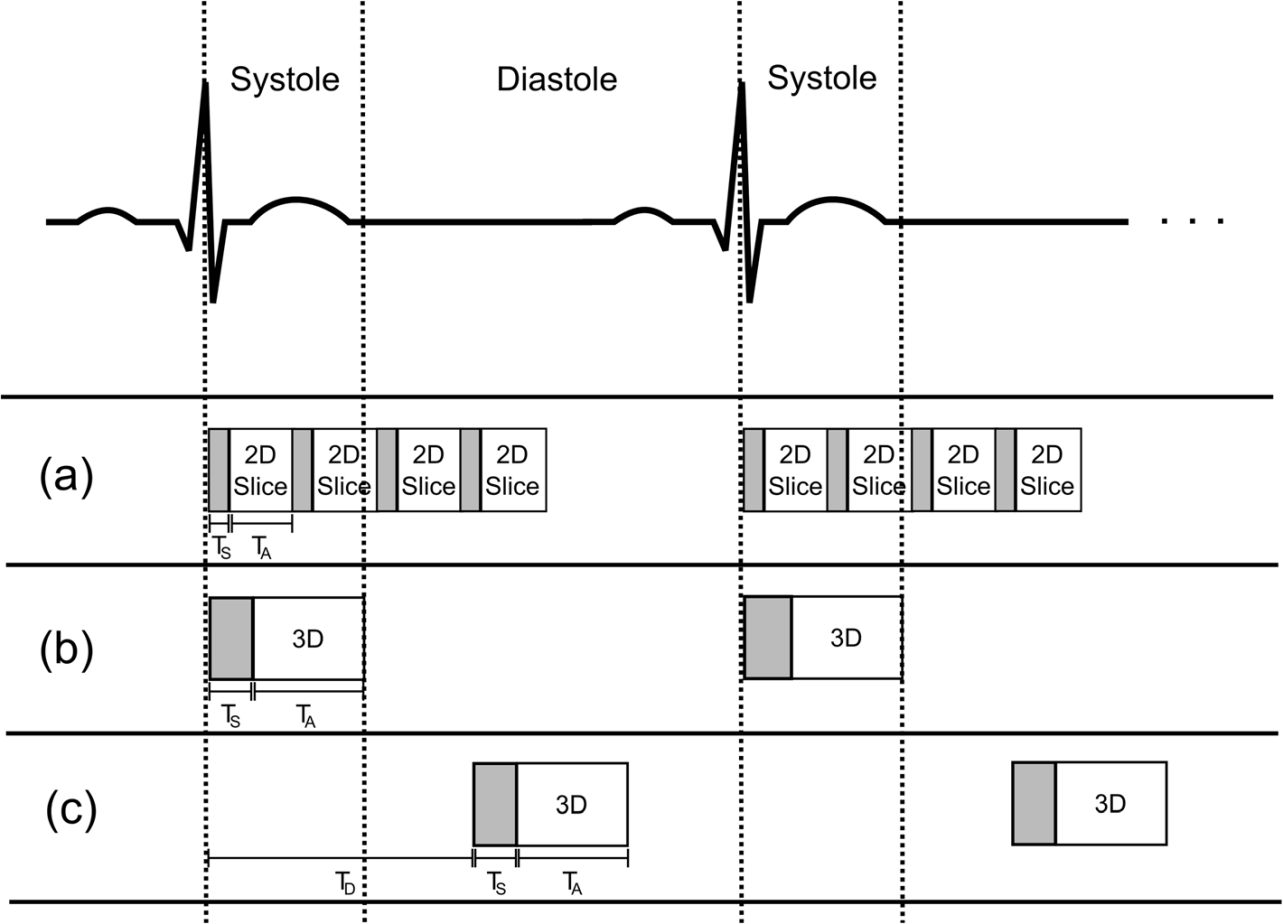
Acquisition timings. Whilst conventional 2D multi-slice FPP is acquired throughout the cycle, typically starting in early systole (**a**), there are two popular options in 3D FPP. The 3D acquisition can be placed either in mid-systole (**b**) or mid-diastole (**c**), although the durations of these periods of minimal motion can be problematic (see text). Trigger delay (TD), saturation time (TS) and acquisition time (TA) are labelled for each in the first cardiac cycle

Whilst the above timings already prevent a temporal resolution of one (or two) cardiac cycles, the cardiac motion itself further limits the acquisition “window” for FPP to either the mid-diastolic or end-systolic pause (Fig. 1). Although some formulations exist for predicting these pause times based on the R-R interval, generally in CMR it is best practice to measure them using cine imaging [17]. For 3D FPP, the end-systolic period has a benefit of the heart being contracted along its long-axis, therefore requiring a smaller 3D partition-direction field-of-view (FOV) which directly reduces the imaging time needed per image. In this phase the myocardium is also transmurally thicker, which has been shown to give greater visualisation of perfusion defects [18]. End-systolic imaging in healthy subjects has been shown in 3D FPP to produce image quality comparable to diastole [19]. The systolic pause is however very short, so a large number of 3D FPP acquisitions to date are acquired in the diastolic pause. During physiological or pharmacological stress both pauses become shorter, particularly at mid-diastole, and so potential acquisition windows are shortened further. Whilst 3D FPP work has a wide range of stated acquisition windows (Table 1), here an ideal time of less than 150 ms for the readout time is used in our estimates. With diastasis durations of ≤100 ms associated with HRs of ≥75 bpm [20], even 150 ms is a compromise towards the longer acquisition windows currently being reported in 3D FPP literature.

The potential utility of 3D FPP has driven a surge in improvement of current acceleration techniques, as well as development of entirely novel processes. Acceleration factors of this magnitude are unlikely to be achieved directly through one method alone. Acceleration methods to date have used a combination of two broad areas: **pulse sequence modification (Section ****Pulse sequence modification****)** and **sub-Nyquist sampling reconstruction techniques (Section ****Acceleration through sub-Nyquist reconstruction****).** This review will focus on the current and potential schemes used in these two areas to attain 3D FPP, along with the difficulties arising from their application and discussion on what the future may hold for this rapidly growing field.

## Pulse sequence modification

Many of the acceleration methods described here can be applied to various CMR pulse sequence types. After a brief recap of the sequences used in FPP, an examination of the potential acceleration techniques will be presented.

### Basic sequence types

Spoiled gradient-echo (SGRE) sequences at low flip-angles are widely used in FPP to produce a steady-state of longitudinal magnetisation, where “spoiling” scrambles or dephases transverse magnetisation to become effectively zero before each RF pulse. Also known as Fast Low Angle Shot (FLASH), these sequences typically further increase the spoiling effectiveness through RF spoiling methods. For SGRE, the choice of flip-angle and the linked impact of B1-inhomogeneity are important in optimising scarce SNR, as reviewed later (Section Alternative k-space coverage).

The transverse magnetisation can effectively be recycled instead of discarded, in the sequence known generically as balanced steady-state free precession (bSSFP, or here as SSFP) [21] which can deliver higher SNR than SGRE as a higher flip-angle can be used. In theory SSFP is a good candidate for FPP; however, increased blood-myocardium contrast in SSFP imaging causes greater artefacts from ringing [22, 23]. The increased flip-angle of SSFP runs into specific absorption rate (SAR) limits, particularly at 3 T, sometimes enforcing a slowdown of the sequence. As well, there is increased unreliability of SSFP at 3 T due to off-resonance effects, whereas SGRE is more robust in regards to both SAR and off-resonance. Despite this, recent work has applied SSFP to 3D FPP at 3 T, investigating use of dual-source parallel transmit capabilities [21]. The application of this technique resulted in SSFP acquired 3D FPP datasets of similar quality to equivalent 3D SGRE datasets, with predicted increased SNR and CNR; however, increased artefacts (including DRAs) were still present.

An extension to SSFP specifically for FPP, called Steady-State First-Pass Perfusion (SSFPP), applies the inherent sqrt(T2/T1) weighting of SSFP in the setting of short native myocardial T2 to deliver myocardial T1-weighting by continuous imaging without saturation pulses [24]. This was presented in 2D FPP as well as showing initial 3D experience. Earlier work used the SGRE steady-state to eliminate saturation pulses [25] with continuous ungated acquisition, before data with similar cardiac phase are identified and reconstructed. Focus was on good SNR and CNR in the myocardium, ignoring effects in the blood pool including inflow artefacts from unsaturated blood, making this implementation of most benefit to non-quantitative FPP. However, for 3D FPP, the acquisition window per 3D image requires further acceleration before this approach may become more realistic.

Almost all 3D FPP work has been based on gated SGRE sequences and the focus of this review will continue with their optimisation.

### Alternative k-space coverage

As with 2D encoding, coverage of k-space in 3D need not necessarily be Cartesian, and two such approaches can accelerate imaging.

**Fig. 2:**
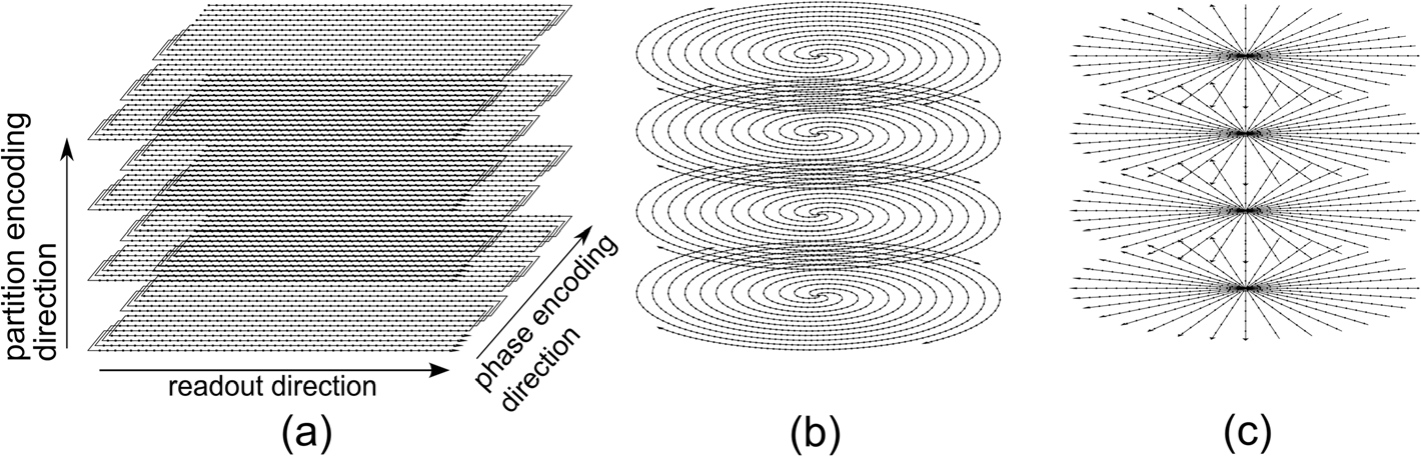
Non-Cartesian trajectories. Examples of three potential alternate trajectories discussed in the text. EPI (**a**) demonstrated with an ETL of 4, a spiral trajectory (**b**) with 4 interleaves and a radial projection design (**c**). Partition encoding direction in (**b**) and (**c**) is the same as for (**a**)

Echo-planar Imaging (EPI) is geometrically closest to typical Cartesian k-space coverage (Fig. 2a) [23]. The premise is to collect multiple ‘lines’ of raw data by a series of gradient echoes after each RF pulse, allowing acceleration by omitting RF excitations. Due mainly to the limited echo-train length (ETL) achievable as a consequence of cardiac motion and main-field inhomogeneities around the heart, hybrid EPI (h-EPI) is generally used in FPP [26] with limited applicability of true single-shot EPI [27, 28]. The compromise for h-EPI is made between increasing the ETL – which reduces total image time – and the corresponding increased unreliability [26, 29] which also increases with main field strength. Despite this, h-EPI in 2D FPP typically uses an ETL of around 4 at 1.5 T (no current examples at 3 T), corresponding to acceleration factors of approximately 2 compared to the FLASH timings calculated earlier (see Appendix). Early examples of 2D FPP with extended LV coverage used h-EPI [26, 30, 31] but so far 3D EPI imaging has largely been limited to non-cardiac work.

Other trajectories in k-space gain their efficiency through altering the geometry of their coverage to collect more data after each RF excitation. Spiral imaging [32, 33] collects data while spiralling outward from the central raw data through k-space (Fig. 2b), replacing the conventional phase-encode and frequency-encode gradients. Again, as with EPI, the multi-shot variants are most commonly used for FPP, due to the relatively large amount of data required and off-resonance effects with long spiral readout durations [34]. Careful choice of readout duration, flip-angle strategy and other characteristics of a spiral sequence have been shown to compensate for spiral related artefacts in FPP to produce high quality images [35]. Extension of spiral sequences to 3D can in theory provide spherical or elliptical coverage [36]. Far more common however is 3D by a stack of spiral planes with Fourier encoding in the third direction, giving a cylindrical distribution. The acceleration achieved with a stack of spiral design for the purpose discussed here is estimated to be similar to that of h-EPI (see Appendix). The spirals can be produced as uniform density, with a constant sampling interval in the radial direction of k-space, or with variable density which utilises greater sampling density radially in the central region of k-space than is used further out. These variable-density spirals have been shown to improve image quality in 2D FPP [37]. Variable density in the radial direction results in either oversampling the centre of k-space to reduce aliasing artefacts [38] or to have undersampled edges combined with a Nyquist sampled centre, suitable for combination with parallel imaging (discussed later). A ‘dual-density’ approach, with uniform fully-sampled centre and uniform undersampled edges of the spirals, was applied by Shin et al. [39] in conjunction with advanced parallel imaging techniques to achieve 3D whole-heart FPP. This gave in-plane resolution of 2.4 mm × 2.4 mm, and compared favourably with the image quality and dynamics of 2D Cartesian acquisitions, with an acquisition time of 230 ms but was performed only at rest.

Sampling with projections (Fig. 2c) through the centre of k-space (‘projection acquisition’ or ‘diametrical sampling’) is now often named ‘radial’ imaging. For Nyquist sampling at the edges of the acquired k-space, radial trajectories massively oversample the centre, leading to high motion robustness. Even when the edges of raw data are undersampled, the full or oversampled centres naturally support parallel imaging and other acceleration techniques. Radial trajectories in themselves are fundamentally somewhat slower than the conventional phase-encoded approach, but they allow acceleration techniques to be applied efficiently. Likely due to its lower comparative efficiency, radial imaging has seen less application to 2D FPP, with its limited applications drawing on its suitability to specific purposes (e.g. multiple samples through the centre of k-space to calculate the arterial input function [40] and its inherent motion robustness for free-breathing [41]). Radial sampling does however lend itself to combination with compressed sensing methods [42] and as greater numbers of motion sensitive compressed sensing methods emerge it may be applied more to 3D FPP [43] (more discussion in Sections Compressed sensing and Motion correction & free-breathing 3D FPP). Recently, radial trajectories have been used for 3D whole-heart FPP sequences [25, 44, 45], combined with compressed sensing. In all cases, a stack of the 2D radial trajectories formed a 3D cylinder, as for the spiral examples. True 3D radial trajectories are also possible [46], with techniques such as Vastly undersampled Isotropic Projection Reconstruction (VIPR) [47] utilising the greater tolerance to undersampling, but these are less common in cardiac work. As with 2D radial, these trajectories are suited to compressed sensing and similar reconstruction techniques [48], however, the small number of projections achievable in 3D FPP and relaxed requirement for isotropic resolution make them less desirable.

Amalgamation of the benefits of the above trajectories can be achieved, for example by adding a spiral twist to the ends of radial projections, known as TWIsting Radial Lines (TWIRL) [49] or using a Cartesian grid acquisition for each projection angle, named Periodically Rotated Overlapping ParallEL Lines with Enhanced Reconstruction (PROPELLER) [50]. These have not been applied to even 2D FPP, but are possible considerations for the highly optimised sequences required for 3D FPP.

### Other k-space efficiencies

**Fig. 3:**
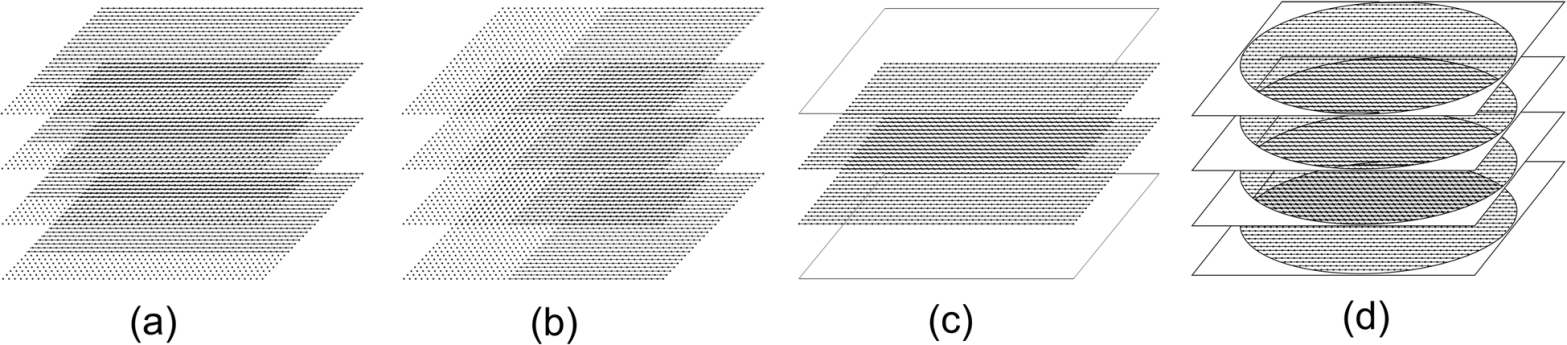
K-space efficiencies. Three k-space acquisition modifications demonstrated for a 3D Cartesian sequence. Representations of partial Fourier in the phase-encoding (**a**) and readout (partial-echo) (**b**) directions are shown, with dashed lines denoting data points that are not acquired but later calculated (see text). Zero padding (**c**) has lines of ‘data’ filled with zeros added either side of the acquired data before reconstruction, artificially increasing resolution. An elliptical shutter (**d**) does not acquire the corner regions of k-space, as they are deemed less critical. Each of these methods can be applied in any encoding direction and those chosen here are simply illustrative

To achieve the acceleration required for 3D FPP, further reductions in raw data coverage are often applied in conjunction with the other methods discussed, although they are compatible with only some non-Cartesian methods (see Section Alternative k-space coverage). These k-space “efficiencies” or ‘tricks’ include methods such as partial Fourier, elliptical shutters, zero padding and zonal-imaging (see Fig. 3) and are described in this section. Many of the details of these techniques can be found in textbooks, such as [51] or [52].

Partial Fourier imaging (Fig. 3a) is also sometimes known as partial averaging (fractional NEX) or partial echo when applied along phase-encode and frequency-encode directions respectively. If fully implemented, partial Fourier uses the “conjugate symmetry” [51] of k-space under certain conditions to reconstruct omitted regions. In reality, phase variations across the image FOV, caused by various factors, break the mathematical conditions supporting this method. In practice therefore, correction can use a low-resolution phase image estimated by raw data collection extending slightly into the omitted half [53–55]. A simpler alternative is more often performed, which is not true partial Fourier reconstruction, simply zero-filling the portions of k-space omitted by the truncated acquisition, for input to the reconstruction by Fourier transformation. However, this usually requires a higher proportion of acquired k-space for acceptable accuracy, and wider Gibbs ringing artefacts are typically provoked by the sudden truncation of sampling nearer central k-space. This process is effectively a filter and consequently reduces spatial resolution. Zero-filling does however allow truncated acquisition to be applied in multiple directions simultaneously (unlike conjugate synthesis), giving greater acceleration. Cartesian examples of 3D whole-heart FPP have used this principle of zero-filled “partial Fourier” extensively, in 2 [56, 57] and all 3 [58] dimensions simultaneously.

Another type of zero-filling or “zero-padding” in raw data operates by “pads” of extra zero-valued lines added symmetrically to both edges of k-space (Fig. 3b) before applying the Fourier transform. This synthetically reduces the pixel size of the reconstructed image, without the added time of acquiring extra data. This is virtually equivalent to post-reconstruction interpolation of pixels, but differs regarding consistency of the Gibbs artefact [59]. This is prominently used in the partition direction in 3D imaging due to the significant extension in time needed to increase the acquired data in this direction, but can be applied in any or all directions. Artefacts such as Gibbs ringing are common with this technique, as previously mentioned, with the sharp cut-off in k-space manifesting as ringing artefacts at strong edges in the image. Some filtering can be applied to reduce this ringing at the cost of loss of resolution. Zero-padding is not always explicitly stated in literature, but with Gibbs ringing a particular issue in DRAs with FPP, care needs to be taken with these techniques.

Another possible efficiency gain, again by omitting regions of k-space, is to exclude the acquisition of the corner regions of k-space (Fig. 3c). This has various names but will here be referred to as an elliptical shutter, due to the typical shape of the acquired k-space afterwards. Due to the small genuine signal amplitude in outer regions compared with the uniformity of noise, removal of the corners of k-space can improve the SNR. Furthermore, with an apodisation filter to reduce Gibbs ringing typically applied in a radial or elliptical fashion for isotropic resolution, the corners of k-space, even if acquired, are typically filtered to zero before reconstruction. Some efficiency can therefore be gained by omitting acquisition of these corners in the first place. However, combination with other efficiencies makes the effects of this process more complex, with the corners of k-space impacting the resolution when applying zero-padding [60]. The combination of an elliptical shutter and 3-dimensional partial Fourier mentioned in the previous paragraph, on top of other acceleration methods (see Section Parallel imaging using joint spatiotemporal redundancy), has resulted in a 3D FPP protocol acquiring just 5 % of the total k-space region [58]. These three types of “zero-filling” k-space efficiency are widely used to further accelerate 3D FPP in addition to the “headline” methods of many papers.

Inner-volume [61], zoomed or zonal-imaging reduces acquisition times by exciting only the required phase-encode FOV, therefore reducing the number of acquired data lines. This can be done by spin-echo, as previously demonstrated in 2D FPP EPI [29, 62], and by two-dimensional spatially selective pulses [63] without spin-echo limitations. The biggest drawback of this latter approach is the complexity and extended duration of zone-selection, which is much slower than ordinary slice-excitation. The scanning efficiency gained by zonal-imaging is therefore highly dependent on the application. For 2D FPP, the phase-encode FOV is typically already minimised to the smallest dimension of the patient’s thorax in the typical short-axis plane, even permitting phase-encode wraparound if it does not reach the LV myocardium. This, combined with the requirement for rapid repetition of RF excitations, limits zone-selective imaging in 3D FPP, based on simple estimates balancing 2D-selective RF pulse duration, ETL, SNR and phase-encode FOV reduction.

## Acceleration through sub-Nyquist reconstruction

The second broad area of acceleration techniques mentioned at the start of this review is sub-Nyquist reconstruction techniques. As with some of the methods discussed above (Section Other k-space efficiencies), they gain efficiency by sampling fewer points in k-space. However, rather than reducing the extent of k-space coverage, these methods accelerate through undersampling, defined as increasing the spacing between k-space samples to an extent that would typically cause intolerable FOV aliasing (wraparound) artefacts [64]. Each version differs in the way undersampling is performed, and also critically the method used to compensate for missing data and reconstruct an image without FOV aliasing. These methods can achieve high acceleration factors and are an essential component in 3D FPP.

### Early work using parallel imaging

One of the biggest breakthroughs in MRI, certainly with regards to imaging acceleration, was the invention and improvement of parallel imaging (PI) methods. The basic premise is to achieve acceleration by utilising spatial redundancy in multiple receiver coils [64, 65] and several varieties are standard on commercial scanners. The ability to perform accurate reconstruction with PI acceleration opened the door to the first attempts at whole-heart FPP. With only relatively low acceleration factors achievable due to the SNR losses accompanying PI, the first adaptations to whole heart coverage used multiple timeframes of FPP series data in calculating coil sensitivity (so called temporal PI techniques) to maximise acceleration.

Köstler et al. used auto-SENSE in 2003 to first demonstrate whole-heart coverage every cardiac cycle, with a contiguous stack of 2D slices [66]. Despite achieving full coverage, only an undersampling acceleration factor of 2 was applied and therefore spatial resolution was coarser than the ideal values considered earlier. Kellman et al. [31] extended the use of h-EPI with TSENSE [67] to produce improved quality in extended coverage FPP, potentially whole-heart, but again limited by the parallel imaging performance to an acceleration factor of 2.

These first two works moved towards whole-heart FPP whilst utilising 2D imaging; the step from 2D to 3D requires greater acceleration. 3D trajectories do, however, allow PI to be split across the two phase-encoded directions [68]. PI performed in this way is more efficient than the same acceleration across just one direction, as increases in the g-factor dependent part of SNR loss can be lower (strongly dependent on coil design). Application of SENSE with an undersampling factor of 6 (3x2, phase-encoded and partition-encoded directions respectively) first demonstrated the feasibility of 3D whole-heart FPP [69]. A more detailed comparison with multi-slice 2D FPP was later made with similar methods but utilising the greater SNR of higher field strengths, and additionally demonstrated the benefit of 3D imaging in estimating defect size [70].

### Parallel imaging using joint spatiotemporal redundancy

**Fig. 4:**
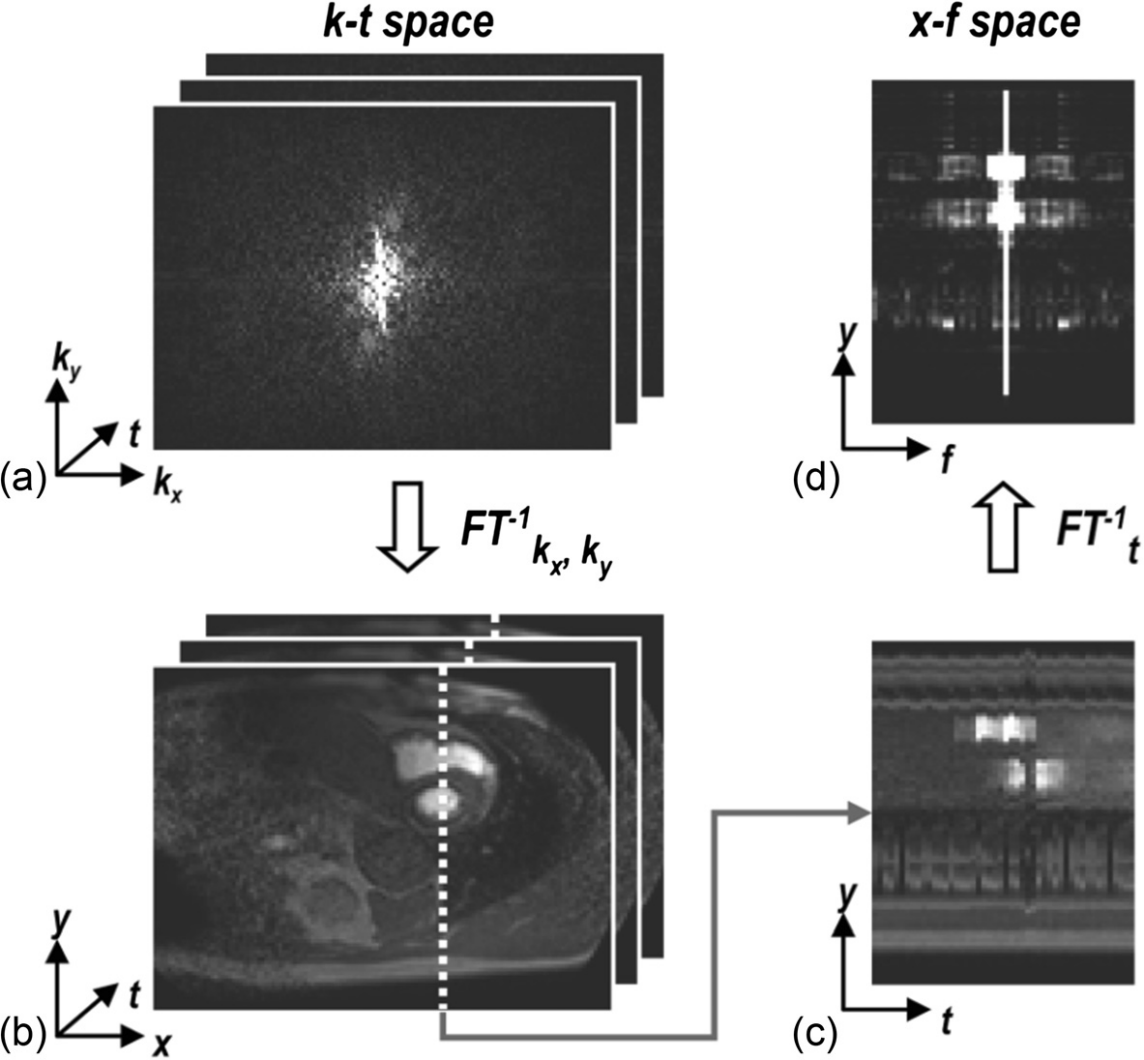
‘Domains’ in FPP. Sets of raw data acquired through time are said to be in k-t space (**a**). Through a Fourier transform in the spatial dimensions this can be converted to a set of dynamic images (**b**), which can be examined for a single line of this data through time (**c**) known as x-t space. A Fourier transform of (c) in the temporal dimension then yields x-f space (**d**). Reproduced from [80]

**Fig. 5:**
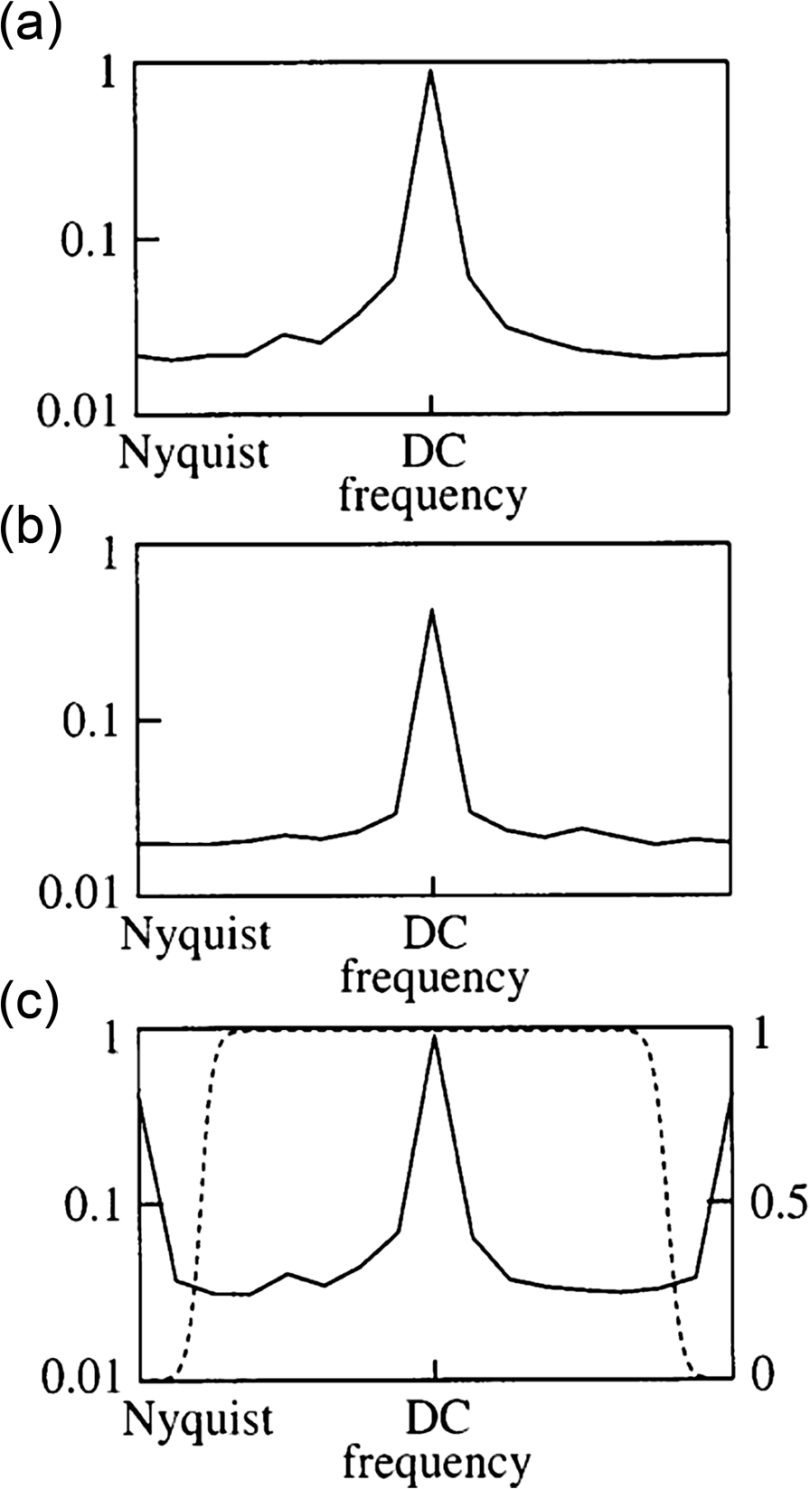
The UNFOLD filter. Example frequency distribution of a more dynamic (**a**) and less dynamic (**b**) region of a cardiac dataset. In the cases of undersampling, resulting in aliasing, the small dynamic region and low undersampling factor allows the unwanted aliasing-induced sidelobes to be removed via a simple filter (**c**). Reproduced from [72]

The feasibility of true 3D whole-heart FPP with good SNR, spatial and temporal resolution improved with the introduction of new PI methods. These take advantage of the similarity of large portions of the images during FPP, and/or the generally gradual changes in image contrast that occur, known technically as using joint spatiotemporal redundancy in dynamically acquired datasets [71]. These techniques are collectively referred to here as k-t PI techniques, due to the temporally (t) varying k-space (k) sampling pattern used in these methods. An extension to the original PI and temporal PI techniques to make simultaneous use of spatial and temporal redundancy, the roots of k-t PI methods can be traced back to the UNFOLD reconstruction algorithm [72]. Redundancy in CMR datasets across time (i.e. across the temporal dimension) can be translated mathematically as a narrower point spread function (PSF) of the series of images when transformed into representation of the different temporal frequencies in the series. This is known as the x-f domain, where x represents all of the spatial dimensions (as with an image in x-y) and f corresponds to frequency, obtained through a Fourier transform across the image in the time series (Fig. 4). This means, with appropriate sampling patterns and small enough acceleration factors, the leakage of PSF energy due to aliasing can be filtered from the true object signal (Fig. 5), which is then Fourier-transformed back to make unaliased images. The process, in effect applying a temporal filter, does not directly cause SNR degradation of gradual changes in image contrast, and therein lies its potential. However, this also ties into a limitation; that more sudden or dynamic real changes in image contrast can lose SNR locally [73], for example if a GBCA bolus remains very compact on arrival in the myocardium.

Whilst UNFOLD and its predecessors uncovered a powerful concept of capitalising on the combined redundancies in spatial and temporal dimensions, its application in cardiac work is mostly limited to acceleration factors of 2 [74] due to the dynamic region being restricted to only 50 % of the FOV. This produces a spreading of the PSF that would overlap at higher acceleration rates and therefore cannot be separated through a simple filter. This minimisation of the dynamic region therefore has a built-in assumption of perfect breath-hold, although a method of easing this constraint to improve applicability to free-breathing FPP has been presented [75].

**Fig. 6:**
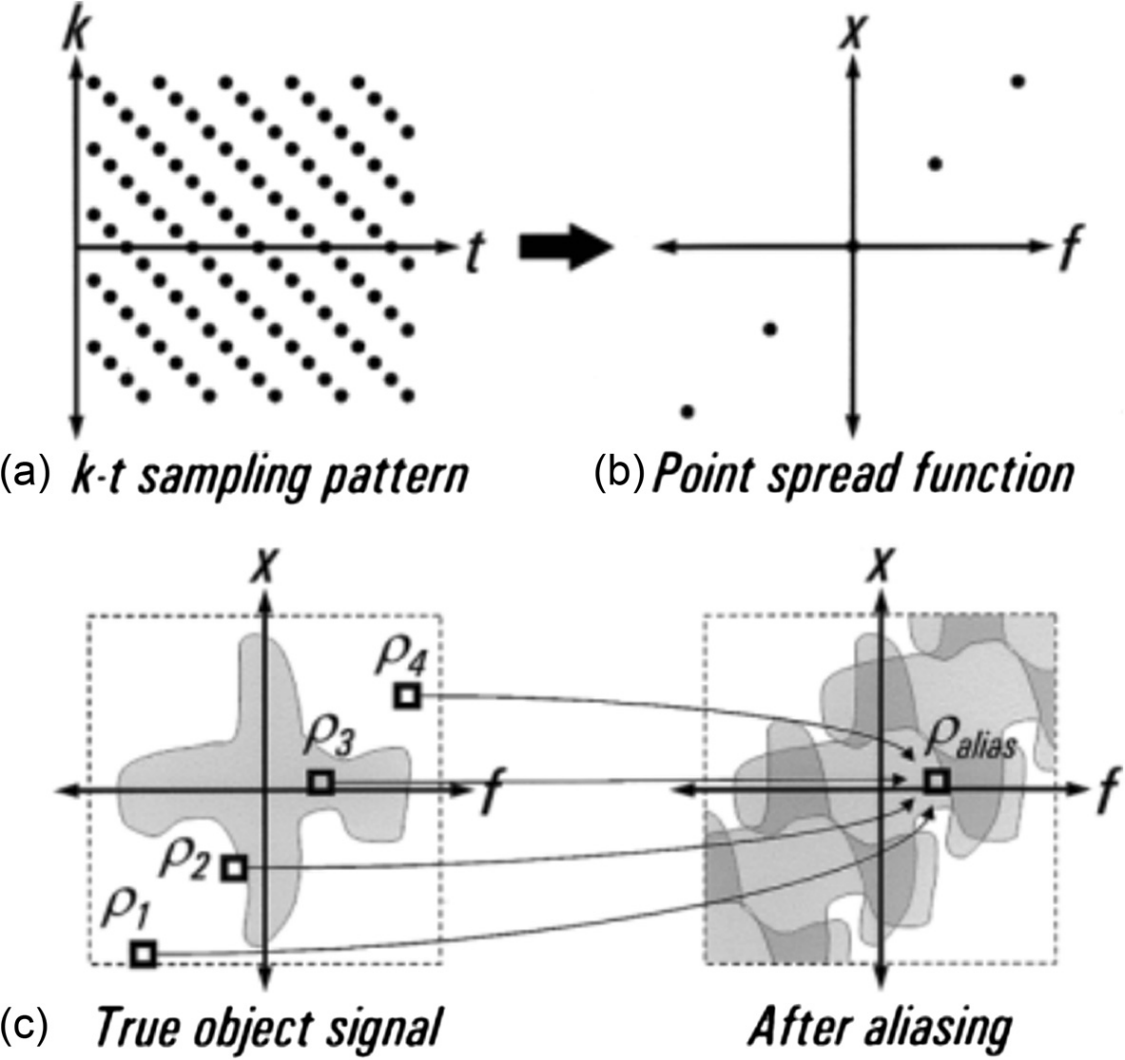
k-t aliasing. With an appropriate undersampling design (**a**) in a FPP series, the distribution of the point spread function (**b**) can be predicted. This gives knowledge of how the true object signal in x-f space (**c**) aliases. Modelling of this predicted overlapping through training data can allow these signals to be separated and therefore permits greater undersampling factors. Reproduced from [76]

Alone this would not support the acceleration required for 3D FPP. Extension to the concept is made through modelling of the expected signal correlations in x-f space using low-resolution unaliased data, known as “training data”. This allows accurate separation of the signal in this space, even for the multiple overlaps resulting from high acceleration factors and dynamic contrast (Fig. 6). This and its enhancement to incorporate parallel imaging are known as k-t BLAST and k-t SENSE respectively [76]. Nominal undersampling factors (undersampling factor, excluding collection of training data) of 5 were demonstrated with k-t SENSE in 2D FPP by Plein et al. [8], with the recouped time used to increase resolution. Vitanis et al. [77] used SENSE to acquire higher resolution training data, which supported a higher undersampling factor (nominal 8, true 5.8) for k-t SENSE accelerated 2D FPP. In theory training data can be collected either through a prescan or integrated into the undersampled data itself each cardiac cycle, although this latter case is by far the most popular. Due to the importance of the training data’s resolution on unwanted temporal filtering effects, an auto-calibrated approach with training data derived from a TSENSE acquisition has also recently been proposed [78] and could be applicable to FPP. k-t BLAST and k-t SENSE have limits in application to FPP due to motion and contrast sensitivity limiting reliability of reconstruction accuracy [8]. As stated earlier, respiratory motion in FPP causes a further spreading of the signal in the x-f domain, beyond the limited spread due to changing image contrast, and therefore such motion reduces the ability of the reconstruction algorithm to correct the aliased data. Despite this, Manka et al. [79] successfully applied k-t SENSE in 3D FPP with a true undersampling factor of 6.3x to a fast Cartesian sequence (including other k-space efficiencies, see Section Other K-space efficiencies), achieving an acquisition window of 200 ms and good spatial and temporal resolution. It has also been applied, using a lower total acceleration, in conjunction with a stack-of-spirals sequence design at similar resolution, though with a longer acquisition window of >300 ms [39].

Transformation of a time-series of images into the x-f domain is the essential component of each of these techniques. Many of the latest techniques aim to accelerate dynamic datasets such as FPP by extending this concept further, with additional or different transformations into mathematical domains that have properties better suiting the reconstruction of the specific dataset type. The method known as k-t PCA is a prime example of this extension and is currently the sub-Nyquist undersampling technique most commonly implemented in 3D FPP literature (see Table 1). As an extension to k-t BLAST (or k-t SENSE), k-t PCA improves the adaptive filter (described above) for removing aliasing while leaving FPP changes unfiltered, by applying principal component analysis (PCA) to the training data used for calculating that filter. This is effectively transforming the images into a new domain of temporal “basis function” components (x-PC) rather than the less suitable temporal pure frequencies as in x-f [80]. The advantage of this principal component domain is that it is more sparse, even in the cases of non-periodic motion such as respiration or misgating. Due to this, the majority of the FPP information is contained within a few principal components, allowing the rest to be discarded. This allows overlapping image space signals to be more easily separated before being converted back into images. Whilst producing large improvements in its ability to cope with greater motion and contrast changes than k-t SENSE, some artefacts and temporal resolution loss can remain in these situations, particularly at higher acceleration factors [73, 81].

**Fig. 7:**
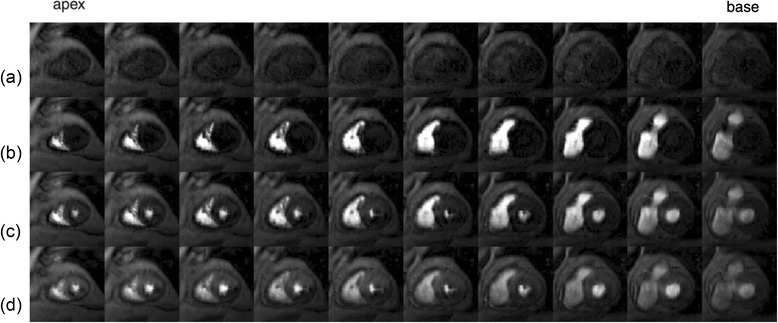
3D whole-heart FPP dataset. An example 3D whole-heart FPP dataset, showing 10 slices before contrast agent arrival (**a**), and during RV (**b**), LV (**c**) and myocardial (**d**) contrast enhancement. The technique used a k-t PI reconstruction technique to enable high levels of undersampling. Reproduced from [81]

Vitanis et al. [81] was the first work to examine the techniques for developing 3D whole-heart FPP with use of k-t PI methods, seen in Fig. 7, by a modified k-t PCA technique designed to support the higher acceleration factors demanded for 3D FPP at higher resolutions. A compartment-based model system was added to k-t PCA using automatic identification of compartments of interest (e.g. LV myocardium, LV blood pool, etc). By using an initial higher-resolution reconstruction process, voxels contaminated by partial volume effects in the low resolution training data could be excluded. This is thought to compensate for a large proportion of the errors in the calculation of the temporal basis functions in conventional k-t PCA. Application of this method allowed temporal and spatial resolution to be maintained (1 RR and 2.3 × 2.3 × 10.0 mm respectively) during whole-heart coverage through use of 10x nominal (5.6-7.5x true) undersampling factor, combined with additional k-space efficiencies. Work from the same group later employed non-rigid motion correction as part of an iterative version of k-t PCA so as to improve 3D FPP reconstruction in the presence of more severe motion, particularly in the case of failed breath-hold/free-breathing [58]. Motion correction increases robustness of the reconstruction scheme to free-breathing or breath-hold failure through frame-to-frame warping of the x-PC training data to match a specified ‘reference’ shape, selected at one phase of the respiratory cycle. Similar acceleration factors and imaging parameters were achieved with this approach, with reported improvement of image quality.

### Other parallel imaging methods

There has been a proliferation in new parallel imaging techniques, based both on spatial and spatiotemporal redundancy, with extensions to the previously described work as well as more unique implementations. While most could in theory be applied to 3D FPP, this review omits much of the parallel imaging work that has not yet been so applied.

CMR reconstruction is fundamentally a “linear” process. The term linear, in situations such as this, simply refers to an output that is proportional to an input, for example, with a tissue of twice the brightness in an image corresponding during scanning to twice the strength of its supporting components of the raw data (k-space) values. PI is also fundamentally a linear process as, using coil response profiles, it solves a set of linear equations. Recent parallel imaging has focussed on exploiting all available data in the most efficient manner. “Self consistency” is one such approach; optimising together (“joint estimation”) the reconstruction of the image and estimation of the coil calibration data, called SPIRiT [82]. When it comes to finding a solution to these types of joint estimation scenarios, the system of equations to be solved no longer linearly connects the raw data to the output images. One way to solve this is “non-linear inversion” (NLINV), repeated inside an iterative search for the best-fit solution [83]. Such a nonlinear scheme with an added variational penalty (described in Section Compressed sensing) has demonstrated high quality reconstructions in real time imaging of the heart, with an acceleration factor of approximately 10 [84]. Nonlinear reconstructions may allow greater reconstruction accuracy for FPP at higher acceleration factors, making it a potential candidate for 3D FPP reconstruction.

Parallel imaging for non-Cartesian trajectories such as radial and spiral can be challenging, as discussed next. The SENSE category of methods, which operate by correcting phase-encode wrap-around in images, are more difficult to apply because the effects of undersampling do not give FOV wrap-around artefacts like Cartesian undersampling. Methods to solve this exist [85], but are not as simple to implement (see Section Computational efficiency). The GRAPPA category, where unsampled raw data is calculated from nearby samples in the raw data, depends on the accurate estimate of the “weighting factors” from sampled to unsampled points; for trajectories over the raw data such as radial and spiral sampling, the estimation of the GRAPPA weights is complicated due to non-equidistant spacing between k-space points. Alternative strategies for calculation of the GRAPPA weights have been proposed to rectify this difficulty for GRAPPA [86–88]. Recently 'through-time' calibration techniques for radial GRAPPA [89] and spiral GRAPPA [90] have been developed, which calculate the weights using multiple fully-sampled prescans. Through-time radial GRAPPA has been used for breath-held 2D FPP achieving whole-heart coverage with 15 slices [91] as well as in 3D for non-FPP applications [92].

Time-resolved angiography With Interleaved Stochastic Trajectories (TWIST), which builds on ‘keyhole’ techniques that update sections of k-space at different rates, alters the 2D phase-encode pattern. The outer portions of the raw data are collected in a pseudo-random pattern that, combined with multiple timeframes, manipulates the undersampling pattern enabling reconstruction via parallel imaging. Originally designed for angiography, it was adapted in SSFPP [24] and used with GRAPPA, as part of a 3D FPP protocol.

Close analogies can be drawn between some of the qualities exploited with these later techniques, namely nonlinear reconstruction and ”random” undersampling patterns, and those used in the final main technique to be discussed – compressed sensing.

### Compressed sensing

This section introduces compressed sensing (CS), followed by the applications of CS to 2D and 3D FPP.

The mathematical framework of CS [93] is relatively recent and was almost immediately considered for CMR due to the inherent suitability of aspects of CMR data. CS utilises the implicit ‘sparsity' in MR images, either in the image itself or in a suitable mathematical representation (i.e. in a transform domain via a ‘sparsifying transform’) of the image, to reconstruct accelerated acquisitions. The term sparsity is simply used to describe a matrix, e.g. of image pixels or raw data points, that are predominately zero valued. Such sparseness may exist after a transform due to redundancy in a single image or over a series of related images. Using this property, CS allows accurate reconstruction of undersampled data, with the proviso that the sampling pattern is ‘random’ and that an appropriate non-linear reconstruction is used [94]. Compression of images using sparsifying transforms is well known [95] and compressed sensing attempts to implement the same concept from the reverse direction; if images can be compressed accurately, then it may be possible to scan faster by acquiring less data in the first place.

The ability to sample a reduced number of random positions in k-space is essential to realise this idea, with such *random* subsampling in k-space resulting in *incoherent* (i.e. noise-like) aliasing artefacts in images. One way to consider this is that strong signals rising above a predetermined threshold can be selected, and the expected interference pattern arising from these signals can be calculated. This interference signal can then be subtracted from the original and the process repeated on the subtracted data, with lowering thresholds, until the true sparse signals have been separated from the aliased signals [42]. In this simplistic version of CS, the process effectively ‘denoises’ the incoherent artefacts created by the random undersampling. In practice, reconstruction of this randomly undersampled data is performed via the solution to an appropriate constrained optimization problem [42].

In MRI, random sampling is limited to phase-encoded direction(s) but can also vary during a series of images, such as during FPP. CS techniques have accelerated 2D FPP [96] for increased resolution or LV coverage, as with k-t PI. CS has an advantage that it does not require training data which can reduce the overall acceleration. Similar temporal characteristics in the reconstructions are seen at lower acceleration factors between CS and k-t PI methods, with the most basic variants of both struggling beyond a critical value of ~5 in human FPP [97]. The increased error for higher accelerations is particularly prominent for CS, limited by insufficient sparsity that is typically achieved in FPP by transformations into other domains, as in k-t PI methods. The x-f domain was popular for early CS work, e.g. k-t SPARSE [98] and k-t FOCUSS [99]. However, in FPP, due to the changing image contrast, a wider range of temporal frequencies causes weaker sparsity in this domain, and therefore other domains have been proposed [100].

**Fig. 8:**
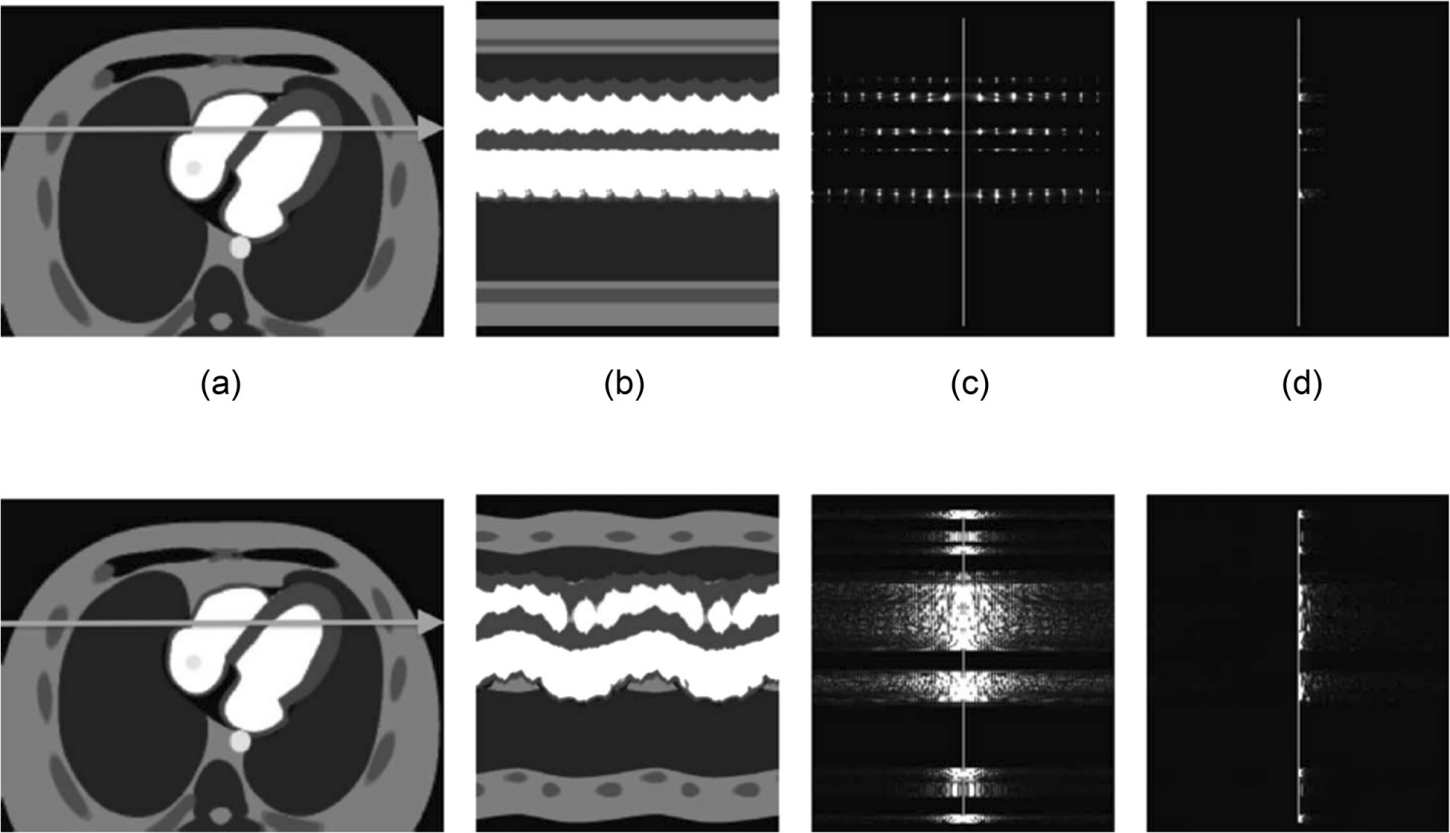
Breath-held and free-breathing sparsity in different domains. The image (**a**), x-t (**b**), x-f (**c**), and x-KLT (**d**) domains of simulated breath-held (top) and free-breathing (bottom) datasets. The x-f domain (**c**) is seen to be far more sparse when the patient is breath-holding than when the patient is allowed to breathe. (**d**) shows the potential for alternate domains to increase sparsity, with improvements in both cases, but of particular importance in the case of free-breathing. KLT stands for Karhunen-Loève transform and is not discussed further - more information can be found in the references in the text. Reproduced from [100]

With potentially high CS acceleration factors under the ideal conditions of good breath-holding and ECG-triggering, work has gone into modifying the standard CS processes to correct for respiratory motion. A technique utilising the Sparsity and Low-Rank properties of the dynamic datasets termed k-t SLR [100] has shown promise in 2D free-breathing FPP in comparison to other CS reconstructions [101], using a transform that provides greater sparsity even in free breathing (Fig. 8). Usman et al. [102] presented free-breathing 2D FPP with more direct motion compensation, improving on methods that adjust for affine deformations (e.g. [99]), integrating a general motion correction technique directly into the CS algorithm. Block LOw-rank Sparsity with Motion-guidance (BLOSM) [103] is another method for motion correction in CS, designed specifically for FPP, combining similar properties of the above methods, dividing the image into regions that can be tracked over time. This was compared with the previously mentioned CS algorithms in 2D FPP under prominent respiratory motion, as well as recent preliminary work in quantitative 3D FPP [104].

Compressed sensing is particularly suited to 3D data (in the case of 3D FPP, a dynamic series of 3D images - “4D” data). The extra dimension(s) and spatial coverage allow greater compressibility of the data. Despite this theoretical advantage, the issues of respiratory motion/misgating mentioned earlier have resulted in limited application of CS to 3D FPP. 3D FPP sequences using CS [25, 44, 45] used temporal and spatiotemporal constrained reconstruction methods from 2D FPP [43]. The first [25] used this reconstruction with ungated imaging (Section Basic sequence types), whilst in [44] it was combined with a more typical radial FPP protocol to achieve good quality images in cases of optimised flip angle. [45] applied a similar reconstruction technique but to a Cartesian SGRE sequence, investigating the effect of trajectory ordering strategy due to imperfect slab excitation profiles (Section Reliability and accuracy).

Recently, a CS algorithm using localised spatiotemporal constraints allowed free-breathing 3D FPP with CS to be demonstrated [105]. Whilst a compartment-based method for k-t PI [81] broke the reconstruction process into compartments of interest, here compartments in the PCA-based sparsifying functions were broken down into smaller patches, allowing overlaps to improve quality, to similarly compensate for differing physiological characteristics during the FPP series. In addition only a subset of the images in the FPP series are considered when, in effect, reconstructing each image - this was hypothesised to suit free-breathing but has the restriction that only moderate motion is expected over a few consecutive frames. This enabled acceleration to an acquisition window of 250 ms at resolution (2.3 × 2.3 × 10.0)mm^3^ and FOV of (340 × 340 × 80)mm^3^ during free-breathing, that compared promisingly against other 3D FPP techniques [105], although was not tested at stress.

Combining parallel imaging with compressed sensing is an intuitive subsequent step and various methods have been proposed [106, 107], as well as CS extensions to joint estimation parallel imaging techniques [84] (see Section Other parallel imaging methods). Otazo et al. [96] applied a combined parallel imaging and CS reconstruction for 2D FPP, and later showed preliminary work in free-breathing, building the motion directly into the sparsity constraints [108]. It seems likely that more reconstruction strategies combining the two will be seen, potentially including 3D FPP.

## Motion challenges

The problems arising from motion in CMR acquisition are well known [109] and FPP is no exception in requiring compensation for cardiac and respiratory induced motion. The necessity for acquiring the data during a quiescent period of the cardiac cycle and the implications of this on the allowed acquisition window were described earlier (Section Requirement for acceleration in 3D FPP). The motion susceptibility of many of the proposed sub-Nyquist reconstruction schemes (Section Acceleration through sub-Nyquist reconstruction) can be particularly problematic in respiratory motion. Furthermore, stress FPP, typically performed through intravenous administration of a pharmacological agent such as adenosine, affects both cardiac [110] and respiratory motion [111]. Whilst motion-correction purely for the improvement of ROI drawing in quantitative perfusion analysis is beyond the scope of this review, issues of breath-hold versus non-breath-hold and cardiac motion directly affect parameters ranging from the required level of acceleration to the final image quality.

### Cardiac motion

Whilst short acquisition windows per slice can be used in 2D FPP, potentially “freezing” motion for the duration of the acquisition, 3D acquisitions require extended acquisition windows within each cardiac cycle, making this assumption of minimal cardiac motion less valid. The largest impact is the introduction of cardiac blurring and DRA effects into the image [112].

Choosing a maximum appropriate acquisition window is difficult, despite its importance in trading off between potential cardiac blurring/reconstruction accuracy and required sequence acceleration. For example, the duration of the typical mid-diastolic quiescence is not only patient specific due to R-R interval, cardiac dysfunctions present, and many other factors, but has been shown to vary (even when normalised to the R-R interval) between cardiac cycles within the same person [113]. The introduction of a pharmacological stressor such as adenosine often increases the heart-rate, further reducing the durations of minimal cardiac movement, particularly in mid-diastole. This all potentially limits the duration of an acceptable typical acquisition window, although there is little literature on this topic in 3D FPP. The stated acquisition windows have varied in 3D FPP literature from 116 ms (with low spatial resolution) up to 380 ms (see Table 1), although some were only performed under resting conditions.

Arrhythmias and ECG triggering unreliability also present challenges, particularly in cases when multiple cardiac cycles are used in the reconstruction (k-t PI and some CS methods) as cardiac phase jitter reduces the temporal sparsity. This somewhat increases the difficulty of routine clinical application. Some transforms are less sensitive to irregular cardiac motion, such that 2D FPP ungated acquisitions have been reconstructed with high quality in patients with atrial fibrillation [114].

### Respiratory motion

It is possible to instruct a patient to breath-hold just before the bolus arrival, and this will usually succeed in providing images of the same myocardial slices during peak enhancement. If an acceleration method depends on breath-hold, it may sometimes be unreliable in a routine clinical environment or where some patients under stress are unable to co-operate with breath-hold instructions. Free-breathing has the drawback for 2D FPP slices that different regions may be seen during the respiratory cycle, another motivation for 3D FPP imaging.

Breath-holding widens the range of sub-Nyquist reconstruction techniques that can be applied. Partial breath-holds, timed to coincide with the myocardial arrival of GBCA, or coached breath-holding may be used, with evident complications in clinical work. It is difficult to repeat FPP scans in the case of a failed breath-hold, and free-breathing robust FPP is therefore a topic of interest.

### Motion correction and free-breathing 3D FPP

With the exceptions of Schmidt [58] and Akçakaya [105], there has been limited progress in making 3D FPP robust to free-breathing. This is owing to the fundamentally severe impact of respiratory motion when using reconstruction strategies that in any sense “share” information from multiple cardiac cycles. The ability to perform 3D FPP with free-breathing, given its dependence on some form of sub-Nyquist sampling, necessitates either mathematical modification to k-t PI/CS algorithms, correction to the data as it is collected or reconstruction strategies that make no use of temporal information.

Modifications to k-t PI methods for free-breathing or motion robustness correct the data or reconstruction into a state similar to some reference respiratory position. However, this involves a distinct step in complexity beyond the non-rigid image warping applied to conventional 2D FPP images [115, 116] or in correcting respiratory drift in a series of single-shot images (as in other applications such as T1-mapping [117]). Such non-rigid “rubber sheet” warping is performed on images that were already completed in separate cardiac cycles, where respiratory motion within the acquired raw data for each image is ignored. However, the modified advanced reconstruction methods must correct respiratory motion during the process of image reconstruction, a much more difficult challenge. This is the case in the “compartmental” and “motion-corrected” k-t PCA methods [58, 81] and adapted CS method [105] applied to 3D FPP discussed earlier, as well as others applied to 2D FPP, e.g. [41, 102, 108]. These are currently popular as they enable the full power, and therefore acceleration, of k-t PI methods.

The challenge with the aforementioned approaches is that they attempt to compensate for raw data that is already ‘corrupted’ by motion; other potential methods may alternatively attempt to collect the data whilst prospectively compensating for the respiratory motion. This can give the additional benefit of correcting through-plane as well as in-plane motion. Diaphragm respiratory position “navigators” are used in CMR [118] for respiratory motion gating or adaptation while scanning. The traditional navigator accept/reject gating cannot be used with FPP because cardiac cycles cannot be omitted. However, the navigator has been used for ‘slice tracking’ the FPP slices to follow the respiratory motion of the heart. This was first demonstrated in FPP by Pedersen et al. [119] and improved with application of a ‘navigator-restore’ pulse to maintain sufficient navigator signal when combined with the FPP saturation recovery sequence [120]. However, as it has to prospectively shift the slice-excitation based on the navigator information, there will always be concerns over its reliability, and a motion-correction model between the typical right-hemidiaphragmatic navigator and the short-axis slices should be employed; this procedure cannot currently be regarded as clinically routine. A similar technique could in theory be used for 3D FPP where slab tracking might be less sensitive to tracking errors.

Finally, more basic undersampling and reconstruction techniques that do not directly utilise the dynamic nature of FPP series would eliminate inter-frame motion sensitivity. These have lower achievable acceleration factors and still require care to ensure that coil calibration methods, or similar, are not affected by respiratory motion. Without using temporal constraints, the challenge shifts for the most part from motion robustness to SNR considerations, due to the fundamental limitations of parallel imaging algorithms at such high accelerations. This could be, for example, the number of spatially significantly different receiver coils required to prevent an underdetermined PI solution. The high acceleration factors required and the prohibitive SNR losses to achieve successful reconstructions with non-k-t PI techniques make this approach less likely to succeed until new methods of accelerating the sequence or improving SNR are realised. Spatially constrained CS could potentially become more important in this way. Additionally, cardiac-specific coil arrays for optimal performance at high acceleration factors [121] would improve high-factor parallel imaging of the LV [122–124]; there are difficulties however in the transfer of these designs to a clinical setting, due to high production cost, variable body habitus, discomfort, or even simply fragility in routine use.

## 3D FPP literature overview

Much of the current 3D FPP literature was discussed above in the context of the advanced techniques used to realise whole-heart coverage. What follows is a more general overview of these evolving protocols, comparing and contrasting the results of these techniques and discussing novelties in their approaches, before taking a more detailed look at their clinical evaluations so far.

### Developmental research review

Through examining Table 1 the development of 3D FPP results is clear, from early low-resolution images acquired at rest first demonstrating feasibility, up to the most recent higher resolution, shorter acquisition window protocols applied to populations with CAD.

The early work by Shin et al. focussed on demonstrating the feasibility and potential benefits of 3D FPP, first [70] comparing with 2D imaging inside an adjustable LV phantom, showing improved accuracy in estimation of defect size, as well as demonstrating in-vivo 3D use and examination of the time intensity curves. The in-vivo experiments included a slice by slice measurement of SNR and CNR in the 3D dataset, which exhibited the predicted losses in the edge slices due to imperfect slab excitation profile, a reason why many later works discard acquired edge slices. Also noticed was flickering in the time intensity curves, predicted to be an effect of the calibration method used in the simple temporal version of parallel imaging applied, as well as significant DRAs due to the low spatial resolution – both indicating need for new acceleration techniques in 3D FPP. The second paper by this group [19] used similar methods for early comparison between systolic and diastolic acquisitions, proposing end-systolic acquisition for 3D FPP in patients with severe arrhythmia. This was done through analysis of time intensity curves in healthy subjects and showed agreement, though increased DRAs were present at the reduced spatial resolution required to image in the shorter period of myocardial stasis in systole.

As previously discussed, the next body of work published made use of k-t PI methods, allowing improvements in many of the protocol parameters. Starting with the clinical application of the sequence [79] (Section Clinical research review) and compartment based improvement to the reconstruction [81], an isotropic in-plane spatial resolution of around 2.3 mm was first achieved and regularly applied thereafter. Alongside the implementation of the compartmental-based adaptation to k-t PCA (from Section Parallel imaging using joint spatiotemporal redundancy) and comparison with conventional k-t PCA using time intensity curves, this paper also examined performance due to number of k-t PCA training profiles and principal components, and various respiratory motion types. However, protocols during a series of clinical research papers [56, 125, 126] and recent examination with parallel transmit [21], continue to use standard k-t PCA without yet including the compartment-based extension. The more recent development of motion-corrected k-t PCA for 3D FPP [58], may possibly require further work before becoming clinically routine. Cartesian-based work from other groups have utilised CS algorithms to investigate motion-sensitivity of the technique [105] and partition ordering effects [45], as discussed in their respective sections.

**Fig. 9:**
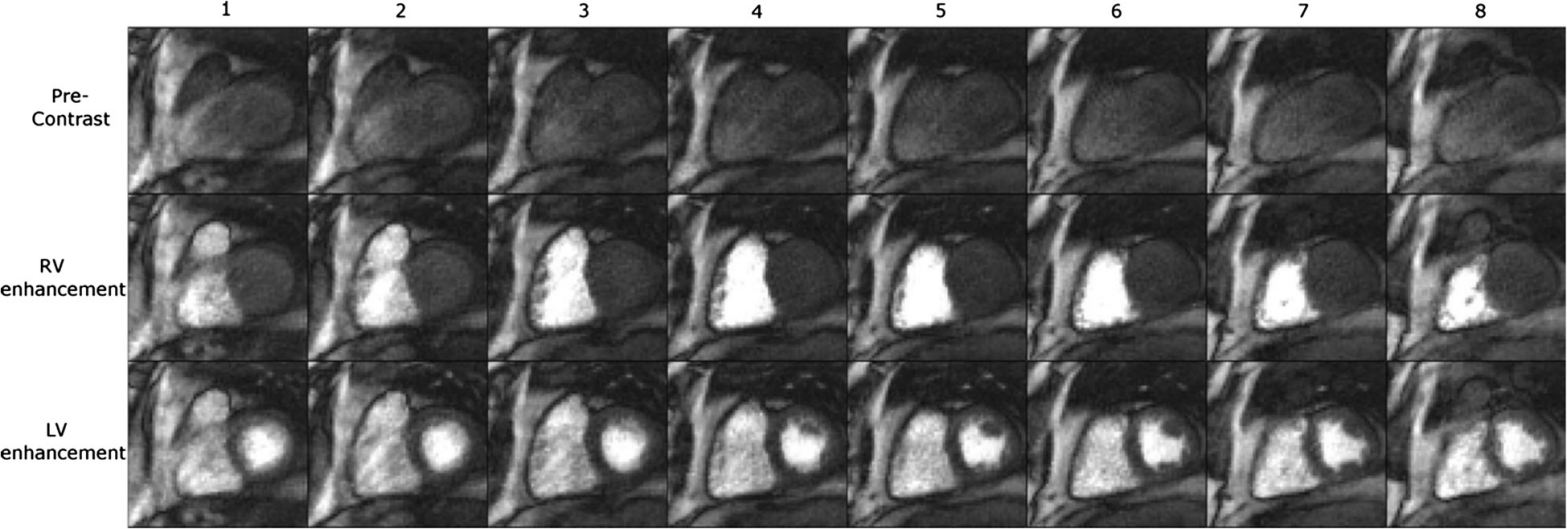
Stack-of-stars 3D FPP dataset. The eight slices of a ‘stack-of-stars’ 3D radial FPP sequence during 3 stages of contrast arrival. Reproduced from [44]

Implementations of 3D FPP through non-Cartesian approaches began with the use of radial sequences, with the first focussing on application of an ungated sequence [25] and the latter testing the feasibility of radial in a more standard gated approach [44] (results in Fig. 9). Alongside numerical simulations to optimise parameters of the sequence as mentioned in Section Alternative k-space coverage, this gated stack-of-stars approach varied the FOV as appropriate, therefore reporting a wider range of resolutions than in other 3D FPP work. With an altered sampling strategy combining higher acceleration and a slightly extended acquisition window, the ungated method gave one of the highest in-plane resolutions so far.

**Fig. 10:**
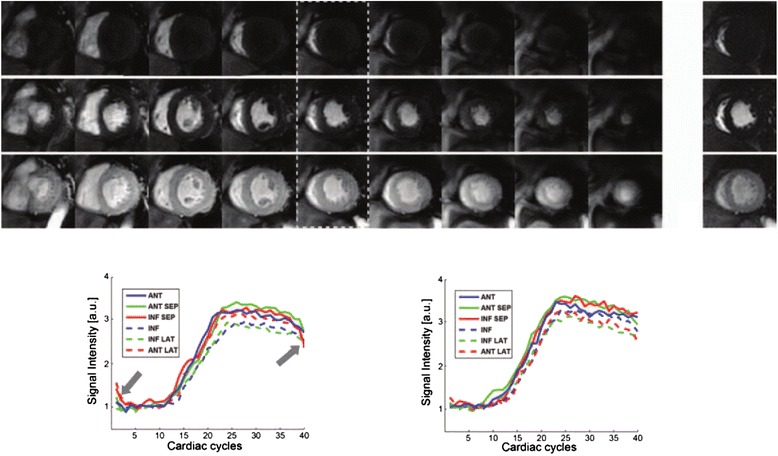
Stack-of-spirals 3D FPP dataset. Illustrative images acquired through stack-of-spirals during right ventricle blood-pool (top), LV blood-pool (middle) and LV myocardial (bottom) enhancement. Dotted lines indicate the middle slice which was used for comparison with the corresponding single-slice 2D Cartesian acquisition. The corresponding myocardial signal-time curves for this and its corresponding 2D slice are shown below, demonstrating good agreement, except for k-t parallel imaging artefacts in the early and late frames. Reproduced from [39]

In work examining the optimisation of the first spiral 3D FPP sequence [39] (see Fig. 10), the higher efficiency of this k-space traversal permitted acquisition windows closer to those applied in the k-t clinical studies, at the smallest undersampling factor applied with a CS/k-t PI technique. With the stack-of-spiral acquisition placed during mid-diastole, a 2D single-slice Cartesian acquisition was also acquired each cardiac cycle allowing comparison of this 2D and 3D approach through analysis of the myocardial signal-time curves (Fig. 10). Finally, alongside the ungated radial approach just mentioned, one of the more novel attempts was continuous acquisition SSFPP [24]. With much of the paper focussing on the SFPP technique in 2D, the 3D initial experience only used a small amount of undersampling and other acceleration techniques, which explains the long acquisition window, nevertheless providing proof of concept.

### Clinical research review

With the widening array of acceleration techniques increasing the feasibility of 3D FPP, a small amount of clinical research has begun. Papers that focus on investigating the clinical potential of 3D FPP rather than protocol design or other topics are examined here in more detail. Table 2 summarises the more clinically relevant aspects of studies containing a population of patients with known or suspected CAD that, among other investigations, compare 3D FPP against a reference standard. The more technical details of the protocols used in these studies can be viewed in their respective entries in Table 1.

**Table 2:** Key parameters of clinical 3D whole-heart first-pass perfusion literature

Lead Author	Year	# of Patients	CMR Centres	CMR Observers	Reference Standards	QCA/%			FFR/%		
						Sensitivity	Specificity	Accuracy	Sensitivity	Specificity	Accuracy
Manka [79]	2011	146	Single	Single	QCA	92 CI[85 99]	74 CI[64 85]	83 CI[76 89]	N/A	N/A	N/A
Manka [125]	2012	120	Dual	Single	QCA & FFR	88 CI[77 95]	75 CI[61 83]	81 CI[73 88]	90 CI[82 98]	82 CI[71 94]	87 CI[80 93]
Jogiya [126]	2012	53	Single	Dual†	QCA & FFR	88 CI[71 96]	80 CI[56 93]	85	91 CI[75 98]	90 CI[66 98]	91 CI[83 95]
Jogiya [57]	2014	45	Single	Single	QCA & MPS‡	94 CI[71 100]	81 CI[54 95]	88	N/A	N/A	N/A
Manka [130]	2015	150	Five	Multiple	QCA & FFR	77 CI[67 85]	94 CI[84 99]	83 CI[76 88]	85 CI[75 92]	91 CI[81 97]	87 CI[81 92]

†3rd observer used when consensus could not be reached

‡For corresponding sensitivity, specificity and accuracy - see text

CI = 95 % confidence interval

In 2011, Manka et al. [79] first investigated the diagnostic ability of 3D FPP, comparing accuracy in identifying significant CAD against quantitative coronary angiography (QCA), as well as demonstrating the potential for volumetry of defect-induced hypointense regions. Compared against QCA, in 146 consecutively recruited patients, the sensitivity, specificity and accuracy were 92 %, 74 % and 83 % respectively, comparing favourably with CMR values in studies using 2D FPP [127, 128]. In a subgroup of 48 patients, who went on to have coronary stenting, repeat stress 3D FPP was performed within 24 h of the procedure. It was in these patients that volumetric analysis of the inducible perfusion defects was performed, with comparison of pre and post procedure showing the predicted large effect of treatment. Further investigations of note, repeated in some of the more recent 3D FPP studies, were on image quality and whole-heart versus 3-slice CMR coverage. Artefacts were split into breathing related, k-t PI reconstruction related and DRA related, which were present in 12 %, 10 % and 8 % respectively. Whilst all images were deemed diagnostic in quality, this relatively high percentage of artefacts, along with their categorisation, highlights many of the problems already discussed in 3D FPP. Examination of 3 slices chosen from the 3D dataset produced a lower sensitivity than for the 3D dataset due to an increase in false-negatives. Whilst this agrees with a predicted advantage of 3D FPP over its 2D counterpart, this method of comparison is not a full test of the two as true 2D acquisitions have different properties, as noted in a later study [57]. That study [57] also included indication of the improved determination of ischaemic burden in 3D over 2D FPP via the same method of using a subset of the 3D dataset’s slices.

With the limitation of poor correlation between the haemodynamic effect of a coronary stenosis and QCA [129], Manka et al. [125] further compared 3D FPP to fractional flow reserve (FFR). The study was also extended to two centres and included a subgroup undergoing repeat examination for inter-study reproducibility, which resulted in excellent correlation. Sensitivity, specificity and accuracy compared to QCA were similar to that in [79], whilst values were improved when using FFR as reference. This trend was also seen in [126], which provided some of the highest accuracy values, using two observers at a single centre and use of the Duke Jeopardy Score to complement FFR. Most recently [130] extension on the dual-centre investigation has been made with a multi-centre evaluation of a similar protocol across five (single vendor) European sites. With 155 patients recruited and 150 successfully examined, this is the largest 3D FPP study to date. With all CMR perfusion analysed in a central laboratory, measured image quality remained good and mean specificity compared against QCA and FFR were the highest of all studies in Table 2, although there was a decrease in sensitivity against the QCA reference.

Also recently [57], the measurement of ischaemic burden by 3D FPP was compared with that by myocardial perfusion scintigraphy (MPS), with sensitivities, specificities and accuracies of the two methods calculated for a subgroup undergoing coronary angiography. These values were 94 %, 81 % and 88 % respectively for CMR 3D FPP and 94 %, 63 % and 79 % for MPS. Comparison of ischaemic burden between the two showed no significant differences. Other clinically focussed work has investigated quantitative 3D FPP, which is of increasing interest [104, 131], including a study of 35 patients that estimated myocardial blood flow and myocardial perfusion reserve in systole and diastole [56].

With all of the clinical studies thus far coming from related centres, parameters are understandably similar. In-plane spatial resolution is 2.3 × 2.3 mm in all cases with through plane resolution being changed from 10 mm to 5 mm after the first study. The first two studies in Table 2 were at 1.5 T, whilst the latest three were at 3 T and state that images were acquired during systole (cardiac phase not described in the first two). Unlike in [79], which used k-t SENSE as the k-t PI reconstruction technique, the latter studies employed k-t PCA. This goes some way to explaining a reduction in the number of k-t PI related artefacts in Jogiya ‘12 and Jogiya’14. Due to the limited variation in implementation, including protocols, reconstruction methods and breath-hold methods (not always stated), extrapolation of results to different CMR systems or non-specialist CMR sites is not yet possible, but these are positive early findings and validate many of the proposed benefits of 3D FPP.

## Future considerations

Various combinations of acceleration methods, that allow total acceleration factors approaching those necessary for 3D FPP have been presented. Further acceleration would be desirable as the image acquisition time within each cardiac cycle remains too long, requiring some care in setup, and the current spatial resolution is also known to be vulnerable to imaging artefacts. Clear evidence for clinical advantages of 3D FPP over 2D would be required before proceeding to expensive large multi-centre, multi-vendor trials. Some of the reviewed 3D FPP methods require improved reliability and computational efficiency before they would be suitable for clinical trials.

### Reliability and accuracy

The highly accelerated dynamic acquisition for 3D FPP is a particularly difficult problem. Some issues lowering the reliability were mentioned for techniques reviewed above, and are addressed further in this section.

Reliability can be an issue with Non-Cartesian trajectories. Such trajectories are a potential approach to 3D FPP sequences, but despite a long period of development there is a scarcity in their routine clinical usage. This can be largely attributed to their reliability depending on extra complications (e.g. requirement of field-map off-resonance corrections, scanner-specific adjustments of sequence timings, and more complex reconstructions) in comparison to what is realistic in a more routine clinical environment. Furthermore, where a long readout is used after each RF excitation, as in EPI and spiral methods, the nominal spatial resolution can be impacted by cumulative off-resonance phase errors during the readout.

Similarly, slice-tracking and other prospective motion-correction algorithms can be unreliable and damage irreparably an acquisition which might otherwise have been of clinically usable quality even if suboptimal. The majority of the presented techniques are in their early research stages and so the consistency of their performance is as yet uncertain. The non-Cartesian work so far has been developmental, applied at rest and requires some evaluation or improvement for stress before even approaching clinical utility. The use of dynamic information to support sub-Nyquist sampling reconstruction schemes has delivered clear improvement in performance. However, study is required over more subtle temporal effects that may arise with these techniques. Temporal smoothing is known in some PI methods that use temporal calibration of coil sensitivity data as well as k-t PI techniques, though methods to improve these issues have been proposed [132]. It is suggested more recent algorithms have shown less of this effect in FPP [80], but careful examination of the effect on dynamic regions of the image in particular may be important in techniques utilising temporal sparsity, even when all of the supporting conditions are perfectly satisfied.

The availability of 3 T, although sometimes of controversial benefit in clinical CMR compared to 1.5 T, is likely to be important for 3D FPP. As many of the acceleration methods sample fewer raw data points, which has a direct effect on SNR, the property of greater SNR at increased field strength becomes desirable. This SNR gain is not always straightforwardly delivered by 3 T for cardiac applications [10, 133]. However, if based on low flip-angles and short sampling trajectories after each RF pulse, especially at peak contrast-agent T1 reduction, as most GBCA relaxivities are not greatly reduced at 3 T vs 1.5 T [134] some SNR enhancement is predictable in 3D FPP. With traditional PI causing reduced SNR proportional to the square root of the acceleration, the increased SNR of higher field strengths can partially compensate. In addition, the coil sensitivity profiles at the higher frequencies of increased field strengths may improve parallel imaging performance (beyond a typical ‘critical limit’ of approximately 4) although this improvement only becomes significant at field strengths typically referred to as ‘ultra-high field’ [135]. Issues with higher field strengths are well known, with increased main field inhomogeneities of particular pertinence for longer raw data sampling after each RF pulse. These may increase some of the unreliability issues in non-Cartesian trajectories, therefore limiting achievable acceleration with these methods, and are responsible for increased artefacts even in standard sequence designs [136]. A comparison of FPP in 2D between 1.5 T and 3 T, using k-t SENSE and other acceleration methods to achieve high spatial resolution acquisitions, showed similar artefacts and diagnostic quality between images acquired at the two field strengths [137].

**Fig. 11:**
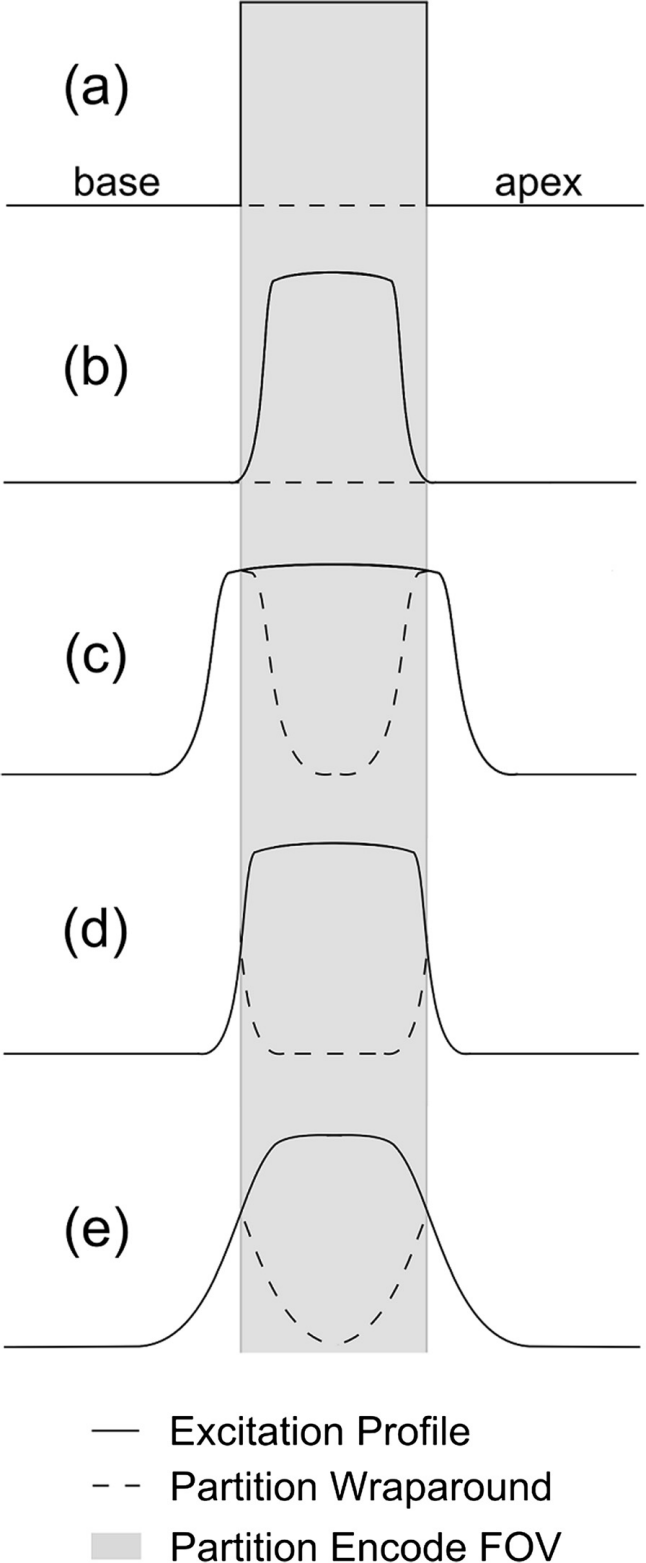
‘Partition-encoding aliasing’ in 3D imaging. Demonstration of partition-encoding aliasing (or “wraparound”) due to slab excitation profile imperfections. An ideal but impossible excitation profile would be as in (**a**), exactly matching the FOV in the slab direction. Using a narrower excitation pulse (**b**) loses SNR in the edge partitions, whilst in (**c**) exciting signal outside the FOV leads to wraparound contamination of many more partitions. A more realistic ideal scenario than (**a**) is shown in (**d**) whereby only the outermost partitions are affected by wraparound and these are usually not displayed. Due to timing constraints in 3D FPP, and therefore the short RF pulses used, avoiding results such as (**e**) is a distinct challenge

As with all 3D CMR the slab profile must be optimised to minimise contamination of the edge partitions (“partition aliasing”) while also exciting sufficient signal towards the edges of the slab (Fig. 11). As in 3D CE-MRA, this problem is exacerbated for FPP because of the fast repeat time of RF excitations, and is commonly concealed by clinical CMR protocols not displaying the edge slices. The 1D-selective slab-excitation RF pulse can potentially be ‘truncated’, enabling a shorter acquisition window within each cardiac cycle by the shorter TE and hence TR of the pulse sequence, as used in [105].

For assessing the clinical reliability of sequences utilising these techniques, large scale clinical trials have shown promise, with good results in large consecutive patient studies (Section Motion challenges). Further studies, including multi-vendor, multi-centre trials, are still required.

### Computational efficiency

Virtually immediate reconstruction of images acquired with standard CMR protocols has become the expectation in the clinical environment. However, the acceleration methods required for 3D FPP demand greatly increased computation for reconstruction, and meeting this expectation of near-immediate results becomes a strong challenge.

Whilst the original version of many parallel imaging and k-t PI methods have an analytical solution, this does not apply to PI methods that involve non-linear components, CS, and some methods modified for motion correction or improved performance. Fundamentally, these methods require a computationally demanding iterative search for the optimised solution (i.e. the images). Further to the choices of search method, of what variables are searched over, and of exactly what types of “constraint” are applied, there are generally also weights controlling how strongly the constraints are enforced, and a stopping criterion for when the search is allowed to conclude (which may in some implementations simply be after a fixed number of iterations). These parameters are clearly crucial to the implementation and affect reconstruction times.

Using non-Cartesian trajectories, regardless of other applied acceleration techniques, also increases the complexity of the reconstruction and with it the computational workload. Gridding prior to fast Fourier transform (FFT), for which various techniques of differing accuracy and complexity exist (very popular is the so-called non-uniform FFT (NUFFT) [138]), is required due to non-uniform spacing of data points in k-space. Trade-offs must often be made between reconstruction accuracy and computational cost, but accurate reconstructions can typically be achieved in reasonable times. When combined with sub-Nyquist methods the reconstructions are further complicated and again require ‘iterative solutions’ as above. This computational strain of advanced reconstructions is especially pertinent for multi-frame 3D FPP sequences with numerous coil channels. For many methods, the reconstruction remains too slow for the images to be viewed while the patient stays in the scanner. Although there may sometimes be little prospect of re-acquiring for other reasons, such slow reconstructions are undesirable and potentially obstructive if a result is poor and an improved re-acquisition is feasible.

Many reconstruction algorithms used in 3D FPP are implemented only in prototype software that requires raw data to be exported from the scanner’s standard clinical software reconstruction system. Such algorithms may be written in MATLAB (MathWorks, Natwick, MA) or similar and are far from optimised in terms of reconstruction time. Whilst this may be acceptable for initial “proof-of-concept” studies, improvements would be required for clinical application, and may be enabled by open-source reconstruction frameworks such as the Gadgetron [139], at least until manufacturers have implemented the more successful approaches.

Many attempts have been made to improve reconstruction times of advanced acceleration algorithms [140–142] and making more efficient use of available hardware such as graphical processing units (GPUs) can reduce reconstruction times [143]. This has been shown for non-Cartesian reconstructions [144] and PI/k-t PI techniques [145], but depends on specialised programming to optimise GPU performance.

## Conclusions

While its clinical utility in comparison to multi-slice 2D remains hypothetical, advances in acceleration methods have opened up the feasibility of achieving 3D whole-heart coverage in FPP. The vast amount of data acquired and the short acquisition window within each cardiac cycle have required the application of multiple techniques simultaneously. Furthermore, the novelty of many of these methods requires further testing of their properties both individually and combined, and few of them are close to routine clinical application, unlike 2D FPP. Challenges with motion remain a real concern, as do reliability and reconstruction times. There is however promise in 3D FPP and with future improvements and careful evaluation of the effects of the applied acceleration techniques, robust 3D FPP may soon be ready for multi-centre, multi-vendor trials to investigate its clinical utility of 3D FPP, both cross-modality and compared with 2D FPP.

## Appendix

Timings of a 3D FPP sequences, given imaging parameters mentioned in Section Imaging parameters for FPP, estimated for typical FLASH, h-EPI and spiral sequences. With no other acceleration techniques applied to reduce these timings, and given the proposed allowed acquisition time, these should allow the reader an idea of approximate relative acceleration achievable through these sequence types and the level of acceleration still required through other methods. Care has been taken to choose values appropriate to the application, but with adjustment of these parameters a topic unto itself the examples provided are clearly only for illustrative purposes.

- T_RF_ – duration of single RF excitation pulse.
- T_RO_ – duration of single ADC readout.
- T_DEAD_ – other time per TR, not due to T_RF_ or T_RO_.

**Table:**

**Desired Parameters**			
FOV/mm		(300 × 225)	
Acquired in-plane resolution/mm		(2.3 × 2.3)	
Acquired phase-encodes, N_Y_		98	
Acquired partition-encodes, N_Z_		12 (to give 100 mm coverage at 10 mm resolution + 1 partition oversampling either side)	
Allowed acquisition window in the cardiac cycle, T_A_/ms		150	
**Calculations**			
	**FLASH**	**h-EPI**	**Spiral**
TR/ms	T_RF_ + T_DEAD_ + T_RO_	T_RF_ + T_DEAD_ + T_RO_	T_RF_ + T_DEAD_ + T_RO_
Total Acquisition Time/ms	TR*N_Y_*N_Z_	TR*N_Y_*N_Z_/N_E_	TR*N_Z_*N_INT_
**Examples**			
T_RF_/ms	0.6	0.6	0.6
T_RO_/ms	0.6	Echo train length, N_E_, dependent. E.g N_E_ = 4 giving 2.4	Number of interleaves, N_INT,_ dependent. E.g. N_INT_ = 16 giving 4.4
T_DEAD_/ms	1.2	1.2	1.2
Total Acquisition Time/ms	2820	1230	1190
Required Acceleration = Total Acquisition Time/T_A_	~19	~9	~8

## Abbreviations

ADC: Analogue-to-digital converter
BLOSM: Block low-rank sparsity with motion-guidance
b-SSFP: Balanced steady state free precession
CAD: Coronary artery disease
CE-MRA: Contrast -enhanced magnetic resonance angiography
CMR: Cardiovascular magnetic resonance
CNR: Contrast to noise ratio
CS: Compressed sensing
DCE: Dynamic contrast enhanced
DRA: Dark-rim artefact
EPI: Echo planar imaging
ETL: Echo train length
FFT: Fast Fourier transform
FLASH: Fast low-angle shot
FOV: Field of view
FPP: First-pass perfusion
GBCA: Gadolinium based contrast agent
GPU: Graphical processing unit
GRAPPA: Generalized autocalibrating partially parallel acquisitions
h-EPI: Hybrid echo planar imaging
MPS: Myocardial perfusion scintigraphy
NLINV: Non-linear inversion
NUFFT: Non-uniform fast Fourier transform
PCA: Principal component analysis
PET: Positron emission tomography
PI: Parallel imaging
PROPELLER: Periodically rotated overlapping parallel lines with enhanced reconstruction
PSF: Point spread function
QCA: Quantitative coronary angiography
RF: Radiofrequency
SAR: specific absorption rate
SENSE: Sensitivity encoding
SGRE: Spoiled gradient echo
SNR: Signal to noise ratio
SPECT: Single photon emission computed tomography
SSFPP: Steady state free precession perfusion
T1: Longitudinal relaxation time
T2: Transverse relaxation time
TWIRL: Twisting radial lines
TWIST: Time-resolved angiography with interleaved stochastic trajectories
UNFOLD: Unaliasing by Fourier-encoding the overlaps using the temporal dimension
VIPR: Vastly undersampled isotropic projection reconstruction
